# Elucidating the Role of Alkali Metal Carbonates in Impact on Oxygen Vacancies for Efficient and Stable Perovskite Solar Cells

**DOI:** 10.1002/advs.202406657

**Published:** 2024-07-25

**Authors:** Won Jin Jang, Eun Ho Kim, Jin Hyuk Cho, Donghwa Lee, Soo Young Kim

**Affiliations:** ^1^ Department of Materials Science and Engineering Korea University Seoul 02841 Republic of Korea; ^2^ Department of Material Science and Engineering Pohang University of Science and Technology (POSTECH) Pohang 37673 Republic of Korea

**Keywords:** alkali metal cation, defect passivation, extended x‐ray absorption fine structure, interfacial engineering, perovskite solar cell

## Abstract

Effectively suppressing nonradiative recombination at the SnO_2_/perovskite interface is imperative for perovskite solar cells. Although the capabilities of alkali salts at the SnO_2_/perovskite interface have been acknowledged, the effects and optimal selection of alkali metal cations remain poorly understood. Herein, a novel approach for obtaining the optimal alkali metal cation (A‐cation) at the interface is investigated by comparatively analyzing different alkali carbonates (A_2_CO_3_; Li_2_CO_3_, Na_2_CO_3_, K_2_CO_3_, Rb_2_CO_3_, and Cs_2_CO_3_). Theoretical calculations demonstrate that A_2_CO_3_ coordinates with undercoordinated Sn and O on the surface, effectively mitigating oxygen vacancy (V_O_) defects with increasing A‐cation size, whereas Cs_2_CO_3_ exhibits diminished preferability owing to enhanced steric hindrance. The experimental results highlight the crucial role of Rb_2_CO_3_ in actively passivating V_O_ defects, forming a robust bond with SnO_2_, and facilitating Rb^+^ diffusion into the perovskite layer, thereby enhancing charge extraction, alleviating deep‐level trap states and structural distortion in the perovskite film, and significantly suppressing nonradiative recombination. X‐ray absorption spectroscopy analyses further reveal the effect of Rb_2_CO_3_ on the local structure of the perovskite film. Consequently, a Rb_2_CO_3_‐treated device with aperture area of 0.14 cm^2^ achieves a notable efficiency of 22.10%, showing improved stability compared to the 20.11% achieved for the control device.

## Graphical abstract

The study explores alkali carbonates (Li_2_CO_3_, Na_2_CO_3_, K_2_CO_3_, Rb_2_CO_3_, and Cs_2_CO_3_), highlighting alkali metal cations' crucial role in suppressing oxygen vacancies. Rb_2_CO_3_ emerges as an optimal interfacial material, boosting perovskite solar cell efficiency to 22.10% with enhanced stability. These valuable insights emphasize the critical importance of alkali metal cation selection in achieving effective and stable perovskite solar cell interfaces.

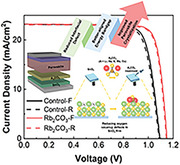

## 1 Introduction

Organic–inorganic halide perovskites, with outstanding optoelectronic properties,^[^ ^1^ ^]^ simple and low‐cost fabrication,^[^ ^2^ ^]^ and an adaptable bandgap,^[^ ^3^, ^4^ ^]^ have been applied in fields such as photovoltaics^[^ ^5^, ^6^ ^]^ and optoelectronic devices.^[^ ^7^, ^8^ ^]^ Perovskite solar cells (PSCs) have achieved a high efficiency of 26.1% within short research periods.^[^ ^9^ ^]^ In particular, FAPbI_3_, which includes a corner‐sharing PbI_6_^2−^ octahedral framework incorporating formamidinium (FA), is widely utilized as the active layer in PSCs because of its ideal bandgap closer to the Shockley–Queisser limit and its thermal stability.^[^ ^10^ ^]^ However, the relatively large ion size of FA leads to tilting of the PbI_6_^2−^ octahedra and lattice distortion, causing instability and deformation to the undesirable *δ*‐phase FAPbI_3_ at room temperature.^[^ ^11^, ^12^ ^]^ Moreover, SnO_2_, commonly employed as the electron transport layer (ETL) in planar PSCs, harbors surface defects such as undercoordinated Sn and oxygen vacancies (V_O_), which induces lattice distortion at the interface and promotes the growth of *δ*‐phase FAPbI_3_ and PbI_2_.^[^ ^13^ ^]^ This, in turn, causes deep‐level trap state defects, leading to nonradiative recombination, which instigates instability along with an open‐circuit voltage (*V*_OC_) deficit.^[^ ^14^, ^15^ ^]^ Therefore, effective passivation of defects at the buried interface, where deep‐level traps predominantly exist,^[^ ^16^ ^]^ is essential for the efficient and stable fabrication of PSCs.^[^ ^17^, ^18^, ^19^ ^]^

Alkali metal salts, commonly employed as SnO_2_/perovskite interfacial materials, simultaneously passivate the interfacial and bulk defects within perovskite films and improve energetic alignment, thereby enhancing efficient carrier transport. Zhuang et al. utilized RbF as an interfacial material and achieved fewer interfacial defects and enhanced electrical conductivity through F–Sn bonding. The Rb^+^, which migrated into the perovskite film, resided at interstitial sites, suppressing ion mobility and demonstrating a low hysteresis phenomenon. As a result, a high efficiency of 23.38% was achieved with a small *V*_OC_ deficit.^[^ ^20^ ^]^ Xu et al. demonstrated that a KF interfacial passivation layer passivates the hydroxyl group defects on the SnO_2_ surface through anion exchange between OH^−^ and F^−^. They also revealed that K^+^ can penetrate the perovskite film, thereby passivating trap state density and nonradiative recombination.^[^ ^21^ ^]^ Zhang et al. compared different Li salts (Li_2_CO_3_, LiC_2_O_4_, and CHLiO_2_); they found that CO_3_^2−^ most effectively suppressed FA vacancy defects, and the residual stress in FAPbI_3_ could be relieved by Li^+^ diffusion.^[^ ^22^ ^]^ However, studies have primarily focused on the properties of anion configurations, neglecting the effects of alkali metal cations and limiting the progress in selecting optimal alkali metal cations for interfacial materials.

In this study, we elucidate the mechanistic role of alkali metal cations (A‐cation; A = Li, Na, K, Rb, or Cs) in interfacial materials and propose a novel and optimal selection of A‐cations. Alkali carbonates (A_2_CO_3_) with different A‐cations were explored to understand the effects of A‐cations on the SnO_2_/perovskite interface, which included an in‐depth analysis to identify the optimal A_2_CO_3_. The interaction of A‐cations with O atoms on the SnO_2_ surface and the formation of single bonds between two O atoms of CO_3_^2−^ and Sn atoms effectively mitigated the formation of V_O_ in the SnO_2_ layer. This not only enhanced the charge‐transfer capability of the SnO_2_ film but also improved the quality of the perovskite film, leading to an enhanced power conversion efficiency (PCE) of PSCs. Combined computation and experimental analysis revealed, for the first time, that the effectiveness of passivating V_O_ defects on the SnO_2_ surface is intricately linked with the size of the A‐cation. Rb_2_CO_3_ treatment achieved the highest efficiency among A_2_CO_3_, along with a significant enhancement in the *V*_OC_. A novel approach utilizing X‐ray absorption spectroscopy (XAS) and grazing incidence X‐ray diffraction (GIXRD) confirmed that Rb_2_CO_3_ at the interface reduces the structural disorder of PbI_6_^2−^ octahedra and residual stress, effectively passivating deep‐level trap states in the perovskite lattice. Concurrently, Rb^+^ diffuses into the perovskite layer, as confirmed by time‐of‐flight secondary ion mass spectrometry (TOF‐SIMS), passivating the defects and increasing the ion migration barriers in the lattice. CO_3_^2−^ also interact with Pb^2+^, providing better crystallinity for both bottom and top perovskite surfaces. As a result, the efficiency of a Rb_2_CO_3_‐treated device with the most favorable energy band alignment increased from 20.11% to 22.10%, thereby addressing issues of low hysteresis and *V_OC_* deficit. Additionally, unencapsulated PSCs demonstrated increased stability both under conditions of high moisture (>50% relative humidity (RH)) with light exposure and at 20% RH under dark conditions.

## 2 Results and Discussion

### 2.1 Elucidating the Role of Alkali Metal Carbonates

**Figure 1:**
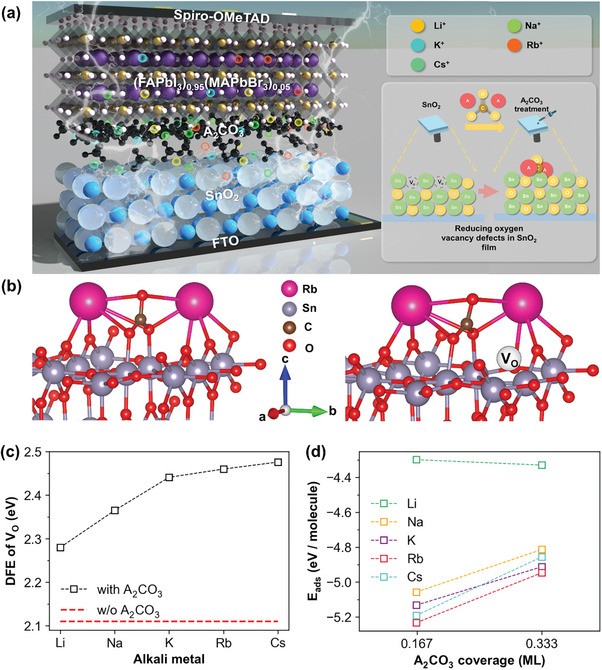
a) Schematic illustration of A_2_CO_3_‐treated PSCs. b) Optimized structure of SnO_2_–A_2_CO_3_ system. The A_2_CO_3_ binds with the surface in the absence of V_O_ (left), with V_O_ (right). c) *DFE*–V_O_ in relation to the type of alkali metal in A_2_CO_3_ at the SnO_2_ surface. The DFE–V_O_ without A_2_CO_3_ is plotted as red dotted line. d) Adsorption energy of A_2_CO_3_ (*E*_ads_) at different surface coverage. Rb_2_CO_3_ has the lowest *E*_ads_ in the given range of surface coverage.

**Figure** **1**a depicts the schematic device structure of the perovskite solar cells of FTO/SnO_2_/(FAPbI_3_)_0.95_(MAPbBr_3_)_0.05_ (MA = methylammonium; CH_3_NH_3_^+^)/*N* ^2^,*N* ^2^,*N* ^2′^,*N* ^2′^,*N* ^7^,*N* ^7^,*N* ^7′^,*N* ^7′^‐octakis(4‐methoxyphenyl)‐9,9′‐spirobi[9H‐fluorene]‐2,2′,7,7′‐tetramine (Spiro‐OMeTAD)/Ag. Various A_2_CO_3_ solutions with different A‐cations were spin‐coated onto SnO_2_ films to stabilize the defects at the buried interface, which presented significantly more deep‐level defects than the bulk perovskite.

First‐principles density functional theory (DFT) calculations were conducted to elucidate the role of A_2_CO_3_ on the SnO_2_ film. A slab system of SnO_2_ (110) was modeled and identified as the most stable surface plane in both experimental and theoretical studies,^[^ ^23^, ^24^ ^]^ as confirmed by the lowest surface energy (Figure S1 and Table S1, Supporting Information).

Subsequently, the defect formation energy (*DFE*) of V_O_ was investigated to determine the impact of A_2_CO_3_ on V_O_ formation, which is known to be a major defect in the performance degradation of SnO_2_‐based PSCs.^[^ ^25^ ^]^ First, the *DFE* of the different V_O_ sites was explored on the surface without A_2_CO_3_ (Figure S2, Supporting Information). The *DFE* of V_O_ at the surface was 1.06 eV lower than that within the subsurface layer, indicating that V_O_ was more readily present at the surface than in the bulk. The interactions between the SnO_2_ surface and A_2_CO_3_ were then examined to determine the change in the *DFE* of V_O_ in the presence of A_2_CO_3_. A single A_2_CO_3_ molecule was affixed to various sites on the surface, revealing a consistent binding pattern (regardless of the type of A), where each A atom interacted with O atoms at the surface, while two single‐bond O atoms in the CO_3_ entity bonded with Sn atoms (the leftmost one in Figure 1b served as a representative example, specifically where A is Rb, while the remaining figure are presented in Figure S3, Supporting Information.). The subsequent investigation of the *DFE* of V_O_ on the A_2_CO_3_‐adsorbed surface confirmed an increase in the *DFE* of V_O_ (the right one in Figure 1b) and demonstrated that the *DFE* increased to 2.28 eV for A = Li and 2.48 eV for A = Cs, compared to the case without A_2_CO_3_ (2.11 eV), as shown in Figure 1c. This suggested that the presence of A_2_CO_3_ at the surface suppressed the formation of V_O_ because the SnO_2_ surface was stabilized by passivation. To determine the increase in the *DFE* of V_O_ with A_2_CO_3_ as the A‐site moved down the periodic table, Bader charge analysis was conducted. The charge analysis showed that A_2_CO_3_ withdrew charge from the SnO_2_ surface, and the transferred charge gradually decreased from 0.198e (A = Li) to 0.157e (A = Cs), as shown in Figure S4 (Supporting Information). The transferred charge reduced the charge on the SnO_2_ surface and weakened the binding between Sn and O at the surface. Thus, as the amount of charge transferred from the surface to A_2_CO_3_ increased, the *DFE* of V_O_ gradually increased. The adsorption energy (*E_ads_*) of A_2_CO_3_ was examined with respect to surface coverage fraction (*θ*) to predict whether the adsorption of A_2_CO_3_ is energetically favorable on the SnO_2_ surface. Figure 1d illustrates that the *E*_ads_ of A_2_CO_3_ was always negative, indicating that the binding of A_2_CO_3_ to the surface was energetically favorable, regardless of surface coverage. However, the binding strength varied with both the A‐cations and the surface coverage. Notably, Rb_2_CO_3_ (red) exhibited the lowest *E*_ads_ for both *θ* = 0.167 and *θ* = 0.333, suggesting that Rb_2_CO_3_ adsorbed most strongly on the SnO_2_ surface. Interestingly, Cs_2_CO_3_ (cyan) showed relatively higher *E*_ads_ than Rb_2_CO_3_ at *θ* = 0.167, and it increased rapidly as the *θ* increased to 0.333. This could be attributed to the large cation size of Cs, which enhances steric hindrance and makes the adsorption of Cs_2_CO_3_ less preferable. In summary, the energetic study indicated that, while the adsorption of Cs_2_CO_3_ most effectively suppressed the formation of V_O_, the large size of Cs introduced steric hindrance, making adsorption less preferable. Consequently, Rb_2_CO_3_ emerged as the best interfacial layer to suppress V_O_ formation on the SnO_2_ surface.

**Figure 2:**
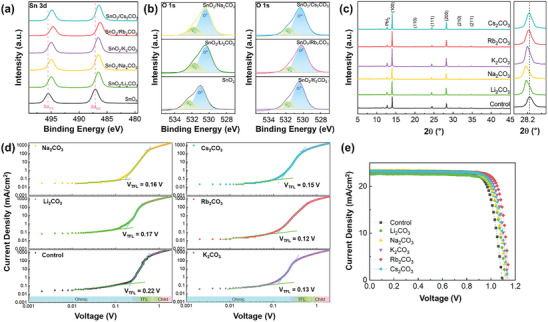
Investigation of the characterization of A_2_CO_3_‐treated films. a) XPS spectra of SnO_2_ and SnO_2_/A_2_CO_3_ films for a) Sn 3d and b) O 1s. c) XRD patterns of perovskite films deposited on SnO_2_ films with and without A_2_CO_3_ treatment, and magnified plots of (200) peaks. d) Space‐charge‐limited current plots of the electron‐only devices with and without A_2_CO_3_ treatment. The trap state density (*N_t_*) was calculated using the formula *N_t_* =  2εε_0_ *V_TFL_*/*eL* ^2^, where *ε* is the relative dielectric constant of FAPbI_3_, *ε*_0_ is the vacuum permittivity, *L* is the thickness of the perovskite film, *e* is the elementary charge, and *V_TFL_* is the trap filled limited voltage. e) *J–V* curves of PSCs with and without A_2_CO_3_ treatment.

The DFT results suggest that the introduction of A_2_CO_3_ with an optimal A‐cation size helps mitigate the formation of V_O_ on the SnO_2_ surface. The chemical interaction between SnO_2_ and A_2_CO_3_ was further confirmed using X‐ray photoelectron spectroscopy (XPS). Upon A_2_CO_3_ treatment, the presence of A‐cation peaks, which are not observed in the typical SnO_2_ layer, indicated the successful binding of A_2_CO_3_ treatment on the SnO_2_ layer (Figures S5 and S6, Supporting Information).^[^ ^26^ ^]^ **Figure** **2**a presents the Sn 3d XPS spectra of the SnO_2_ films with and without A_2_CO_3_ treatment. The Sn 3d_5/2_ and Sn 3d_3/2_ binding energies shifted slightly to lower values upon A_2_CO_3_ treatment (Table S2, Supporting Information), suggesting a modification in the electron cloud density surrounding the Sn atom.^[^ ^27^ ^]^ Notably, the Rb_2_CO_3_‐treated film exhibited a more pronounced shift than the Cs_2_CO_3_‐treated film, suggesting a stronger interaction with SnO_2_, consistent with the lowest *E_ads_* in the DFT results. The O 1s peak in Figure 2b can be deconvoluted into lattice oxygen (O^2−^) with an Sn─O bond at 531 eV and V_O_ on the SnO_2_ surface at 532 eV.^[^ ^28^ ^]^ With the shifts toward lower binding energies, quantitative peak area analysis of the O^2–^ and V_O_ peaks revealed a decrease in the area of V_O_ after A_2_CO_3_ treatment. Evaluating the ratio of the peak area of O^2–^ to V_O_ revealed that, in the control SnO_2_ film, the area ratio of V_O_ was 28%, whereas, with Li_2_CO_3_, Na_2_CO_3_, K_2_CO_3_, Rb_2_CO_3_, and Cs_2_CO_3_ treatments, it decreased to 26%, 23%, 20%, 15%, and 20%, respectively, suggesting a reduction in surface V_O_ acting as electron trap states.^[^ ^29^ ^]^ This implied that the C─O bonds of A_2_CO_3_ and undercoordinated Sn mutually created coordinated bonds, forming A–O–Sn bonds and effectively filling the V_O_ on the surface of the SnO_2_ film. The interaction energy between A_2_CO_3_ and V_O_, calculated using DFT (refer to Figure S7, Supporting Information), supported the facilitation of V_O_ filling.

To investigate the electrical properties of the SnO_2_ films after A_2_CO_3_ treatment, their dark current–voltage (*I–V*) characteristics were compared (Figure S8, Supporting Information). The conductivity of the control SnO_2_ film was 2.54 × 10^−3^ mS cm^−1^, and that of the SnO_2_ films treated with A_2_CO_3_ was enhanced, as shown in the inset of Figure S8 (Supporting Information). The improved charge‐transfer capability of the SnO_2_ film with A_2_CO_3_ treatment could be attributed to the reduction in V_O_ defects through the formation of A─O─Sn coordination bonds and the enhanced electrical dipole moment at the interface.^[^ ^30^, ^31^ ^]^ Specifically for the K‐, Rb‐, and Cs‐type elements, stronger interactions with the SnO_2_ film and robust V_O_ suppression led to a more substantial improvement in conductivity. The optical transmittance spectrum of the SnO_2_ films on FTO with A_2_CO_3_ treatment (Figure S9, Supporting Information) indicated consistently high transmittance across wavelengths, ensuring efficient light absorption capability into the perovskite layer.

To investigate the effect of A_2_CO_3_ treatment on the crystal structure of the perovskite films, X‐ray diffraction (XRD) and field emission‐scanning electron microscopy (FE‐SEM) were used to examine the perovskite films deposited on various SnO_2_ substrates. As shown in Figure 2c, the additional peak observed at 12.7° in all perovskite films indicated the presence of an over‐stoichiometric PbI_2_ crystal phase, which led to instability and large hysteresis of the devices.^[^ ^32^ ^]^ A_2_CO_3_ treatment suppressed the intensity of the PbI_2_ peak (Figure S10, Supporting Information), which was possibly related to the reduction of V_O_ at the interface.^[^ ^13^, ^33^ ^]^ Figure S11, Supporting Information, also illustrates the reciprocal of full width half‐maximum of (100) peak in perovskite films, showing narrower diffraction peaks ranging from 10.95 to 14.79 *θ* ^−1^ with A_2_CO_3_ treatment. The modified ETL, specifically for K‐, Rb‐, and Cs‐type elements, led to an increase in the peak intensity without the formation of a new phase compared with the control film, indicating that A_2_CO_3_ treatment induced high‐quality perovskite films. Based on the magnified (200) diffraction patterns in Figure 2c, the A_2_CO_3_‐treated perovskites, excluding Cs_2_CO_3_, exhibited lower 2*θ* shifts in their diffraction peaks, indicating interstitial occupancy due to the diffusion of A‐cations into the perovskite films.^[^ ^34^ ^]^ Alkali metal cations, with their high diffusion tendency and low binding affinity to perovskite, easily migrate toward the perovskite film at the SnO_2_/perovskite interface.^[^ ^20^, ^35^, ^36^, ^37^, ^38^, ^39^ ^]^ Cs^+^ with a relatively larger ion size was the only one without a significant decrease in 2*θ*, suggesting a higher probability of distributed Cs^+^ replacing A‐sites rather than occupying interstitial sites. The incorporation of small amounts of A‐cations into perovskite films is anticipated to contribute to elevating the migration energy barrier for native halide defects, thereby controlling defects and acting as a stabilizer for the black phase, which would ultimately lead to the formation of high‐quality perovskite films. The top‐view SEM images in Figure S12 (Supporting Information) visually confirm that A_2_CO_3_ treatment contributes to enhancing the quality of perovskite films and increasing the average grain size, which is consistent with the XRD results. We further investigated the Urbach tail of the perovskite films to evaluate the structural disorder and deep energy levels at their band edge using their ultraviolet‐visible (UV–vis) absorption spectra (Figure S13, Supporting Information). As shown in Figure S14 and Note S1 (Supporting Information), the control film exhibited the highest Urbach energy (*E_U_*) of 28.47 meV, while the Rb_2_CO_3_‐treated film achieved the lowest *E_U_* of 23.98 meV, indicating effective passivation of deep‐level defects at the band edge. This suppressed non‐radiative recombination and ultimately enhanced the *V_OC_* efficiency.

To investigate the trap density of the perovskite films with A_2_CO_3_ treatment in the entire device, we fabricated an electron‐only device (FTO/SnO_2_/perovskite/[6,6]‐Phenyl C61 butyric acid methyl ester/Ag) and conducted space‐charge‐limited current measurements. In Figure 2d, the expected *N_t_* values of the A_2_CO_3_‐treated devices are lower than that of the control device (3.40 × 10^5^ cm^−3^), with the value being highest for the Li_2_CO_3_‐treated device (2.62 × 10^5^ cm^−3^), followed by the Na_2_CO_3_‐ (2.47 × 10^5^ cm^−3^), Cs_2_CO_3_‐ (2.31 × 10^5^ cm^−3^), K_2_CO_3_‐ (2.01 × 10^5^ cm^−3^), and Rb_2_CO_3_‐ (1.85 × 10^5^ cm^−3^)‐treated devices. This demonstrated that A_2_CO_3_ treatment is effective in reducing trap‐assisted Schockley–Read–Hall (SRH) recombination. Consequently, the Rb_2_CO_3_ treatment exhibited the lowest value, indicating the most effective passivation of the electron trap density and the defect density of the perovskite layer upon Rb_2_CO_3_ treatment, aligning with the largest grain size in the FE‐SEM results.

To gain insight into the beneficial defect passivation of A_2_CO_3_ treatment at the SnO_2_/perovskite interface, the photovoltaic performances of the devices with aperture area of 0.14 cm^2^ were screened at various concentrations (Figure S15, Supporting Information). Figure 2e and the corresponding parameters in Table S3 (Supporting Information) present champion *J–V* curves of PSCs with different A_2_CO_3_ treatments at the SnO_2_/perovskite interface. The control PSCs with a pristine SnO_2_ ETL exhibited a relatively low *V_OC_*, yielding a *PCE* of 20.11% with a short‐circuit current density (*J_SC_*) of 23.22 mA cm^−2^, *V_OC_* of 1.093 V, and fill factor (*FF*) of 79.27%. Upon A_2_CO_3_ treatment, the device performance was improved through the modification of the interface between SnO_2_ and perovskite, regardless of the A‐cations used, as shown in Table S3 (Supporting Information). Analysis of the statistical distribution of the performance parameters for different A_2_CO_3_‐treated SnO_2_ films, as shown in Figure S16 (Supporting Information), revealed a gradual increase in efficiency in the order of Li_2_CO_3_ < Na_2_CO_3_ < Cs_2_CO_3_ < K_2_CO_3_ < Rb_2_CO_3_, consistent with the aforementioned results. This improvement, primarily attributed to an improvement in *V_OC_*, could be attributed to the effective passivation of V_O_ and the enhanced quality of the perovskite film, which led to suppressed non‐radiative recombination at the SnO_2_/perovskite interface. Additionally, Figure S17 (Supporting Information) presents the external quantum efficiency (EQE) spectra and the integrated *J_SC_*, showing a good correlation with the enhanced *J_SC_* observed in devices treated with A_2_CO_3_. Consequently, A_2_CO_3_ treatment at the SnO_2_ and perovskite interface had a positive impact, primarily contributing to the improvement of *V_OC_*, with the efficiency tending to increase with increasing size of the A‐cation. Notably, Cs_2_CO_3_, which had the largest ion size and exhibited a slight decrease in efficiency, induced steric hindrance, leading to an inefficiency in stabilizing the SnO_2_ surface compared to the optimal ionic size of the A‐cations. Among the A_2_CO_3_‐treated devices, the Rb_2_CO_3_‐treated device demonstrated a remarkable efficiency improvement of 22.10%, mainly attributed to the *V*_OC_ increase from 1.10 to 1.15 V, revealing the importance of an optimal size of A‐cations in the interfacial materials of PSCs.

### 2.2 In‐Depth Analysis of Optimal Alkali Carbonate in Perovskite Solar Cells

**Figure 3:**
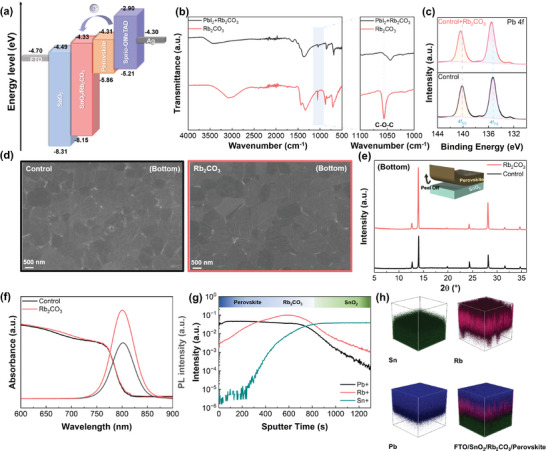
Investigation of the characterization of Rb_2_CO_3_‐treated devices. a) Schematic illustration of energy level diagrams of control and Rb_2_CO_3_‐treated PSCs. b) FTIR spectra of Rb_2_CO_3_ and Rb_2_CO_3_ with PbI_2_. c) XPS spectra of perovskite films with and without Rb_2_CO_3_ for Pb 4f. d) Top‐view SEM images of the buried interface of control and Rb_2_CO_3_‐treated perovskite films. e) XRD patterns of the buried interface of perovskite films SnO_2_ films with and without Rb_2_CO_3_ treatment. f) UV–vis and steady‐state PL spectra of control and Rb_2_CO_3_‐treated perovskite films. g) TOF‐SIMS curves of FTO/SnO_2_/Rb_2_CO_3_/perovskite films, and (h) 3D distributions of Sn, Rb, Pb elements from TOF‐SIMS results.

To understand the impact of Rb_2_CO_3_ on the energy levels of the SnO_2_ film, Tauc plots from UV–vis absorption and ultraviolet photoemission spectroscopy spectra were obtained (Figures S18–S20, Supporting Information). As shown in **Figure** **3**a, the reduced energy loss between the conduction band of the SnO_2_ and perovskite, attributed to the dipole moment of Rb_2_CO_3_, led to a reduction in non‐radiative recombination and charge accumulation at the SnO_2_/perovskite interface. This resulted in an improved *V_OC_* and FF.^[^ ^40^, ^41^ ^]^ In addition to analyzing Rb_2_CO_3_, the effects of other A_2_CO_3_ treatments on SnO_2_ film energy levels were explored, as shown in Figure S21 and S22 (Supporting Information). Notably, Rb_2_CO_3_‐treated SnO_2_ film exhibited the closest alignment of its conduction band minimum with the perovskite film compared to other A_2_CO_3_‐treated SnO_2_ films, which is beneficial for electron transport at the SnO_2_/perovskite interface. Moreover, the characteristics of the SnO_2_ films of Rb_2_CO_3_ treatment were further analyzed to reveal the effect of Rb_2_CO_3_ at the interface (Figure S23, Supporting Information). The water contact angles of both the SnO_2_ and SnO_2_/Rb_2_CO_3_ films demonstrated enhanced hydrophilicity, decreasing from 27.26° to 16.87° upon Rb_2_CO_3_ treatment (Figure S24, Supporting Information). Additionally, the lower roughness (Figure S25, Supporting Information) and surface tension of SnO_2_/Rb_2_CO_3_ films contributed to improved crystallinity of the perovskite films, as shown in Figure S26 (Supporting Information). To assess the impact of Rb_2_CO_3_ deposited on SnO_2_ films at the interface of perovskite films, Fourier transform infrared (FTIR) and XPS analyses were carried out. Figure 3b presents the FTIR spectra of pure Rb_2_CO_3_ and Rb_2_CO_3_ with PbI_2_, revealing a noticeable shift in the C─O─C stretching vibration from 1055.89 to 1044.09 cm^−1^ upon mixing with PbI_2_, indicating the interaction between CO_3_^2−^ from Rb_2_CO_3_ and PbI_2_. Additionally, Figure 3c shows the XPS spectra of Pb 4f in the perovskite film with and without Rb_2_CO_3_, indicating a shift toward higher binding energies from 135.26 to 135.49 eV for Pb_7/2_ and from 140.11 to 140.34 eV for Pb_5/2_. These collectively suggest the presence of interactions between undercoordinated Pb and CO_3_^2−^ from Rb_2_CO_3_, acting as a successful passivation of undercoordinated Pb defects at the interface.^[^ ^42^ ^]^ To further investigate the impact of Rb_2_CO_3_ treatment on the SnO_2_/perovskite interface, we exposed the buried interface of the perovskite films by removing them from the substrate, as depicted in Figure S27 (Supporting Information). The top‐view SEM images and XRD patterns of the buried interface of the perovskite films are shown in Figure 3d,e. The bottom surface of perovskite films shown in Figure 3d exhibits improved crystallinity with a denser and more uniform morphology compared to control perovskite film. XRD analysis of bottom surface of perovskite was also conducted to examine effect of Rb_2_CO_3_ on upper perovskite, as illustrated in Figure 3e. Notably, the XRD patterns of Rb_2_CO_3_‐treated bottom surface show a similar trend to that of the top surface, with a decrease the PbI_2_/(100) peak ratio from 0.2540 to 0.1106 and an increase in the (100)/(200) peak ratio from 2.2354 to 2.4740 compared to control film. These improvements in film quality at the interface can attributed to the decreased presence of V_O_ in SnO_2_ films and interaction between CO_3_^2−^ with undercoordinated Pb. Defect‐induced carrier recombination losses at the interface were investigated based on the optical characteristics of the perovskite films. The intensity of steady‐stated photoluminescence (PL) spectra in Figure 3f for the perovskite film increased upon Rb_2_CO_3_ treatment without a shift in the peak position. This could be attributed to the reduction in charge carrier recombination resulting from the reduced defects and structural disorder in the perovskite films, which is consistent with the UV–vis, XPS, and SEM results. The time‐resolved PL (TRPL) results in Figure S28 (Supporting Information) further demonstrate an increased average carrier lifetime for the Rb_2_CO_3_‐treated film compared with that of the control, indicating the effective control of interfacial trap sites by Rb_2_CO_3_.^[^ ^43^, ^44^, ^45^, ^46^ ^]^ Consequently, the enhanced PL, TRPL, and fluorescence lifetime imaging (Figure S29, Supporting Information) results for the perovskite film with Rb_2_CO_3_ treatment demonstrate the effective passivation capabilities of the interfacial trap sites.

To explore the behavior of A‐cation in Rb_2_CO_3_, we measured depth profiles using TOF‐SIMS. Figure 3g,h illustrates the vertical distribution of the main elements (Sn, Rb, and Pb) in the FTO/SnO_2_/Rb_2_CO_3_/perovskite film and the corresponding 3D ion distribution images. The curve of Rb^+^ versus the sputtering time confirmed their broad distribution, indicating their presence not only at the perovskite/SnO_2_ interface but also within the perovskite layer. As shown in Figure S30 (Supporting Information), the FE‐SEM images of the perovskite films using a high concentration of the Rb_2_CO_3_ precursor further provide evidence of Rb^+^ penetration into the perovskite. The diffusion of a small quantity of Rb^+^ into the perovskite layer is characterized by its partial incorporation at the interstitial sites in the grain boundaries, effectively increasing the halide vacancy formation energy, which enhances structural stability. Additionally, Rb^+^ plays a role in inhibiting ion diffusion migration, thereby passivating the leakage current and contributing to the improvement in *V_OC_*.^[^ ^35^ ^]^

**Figure 4:**
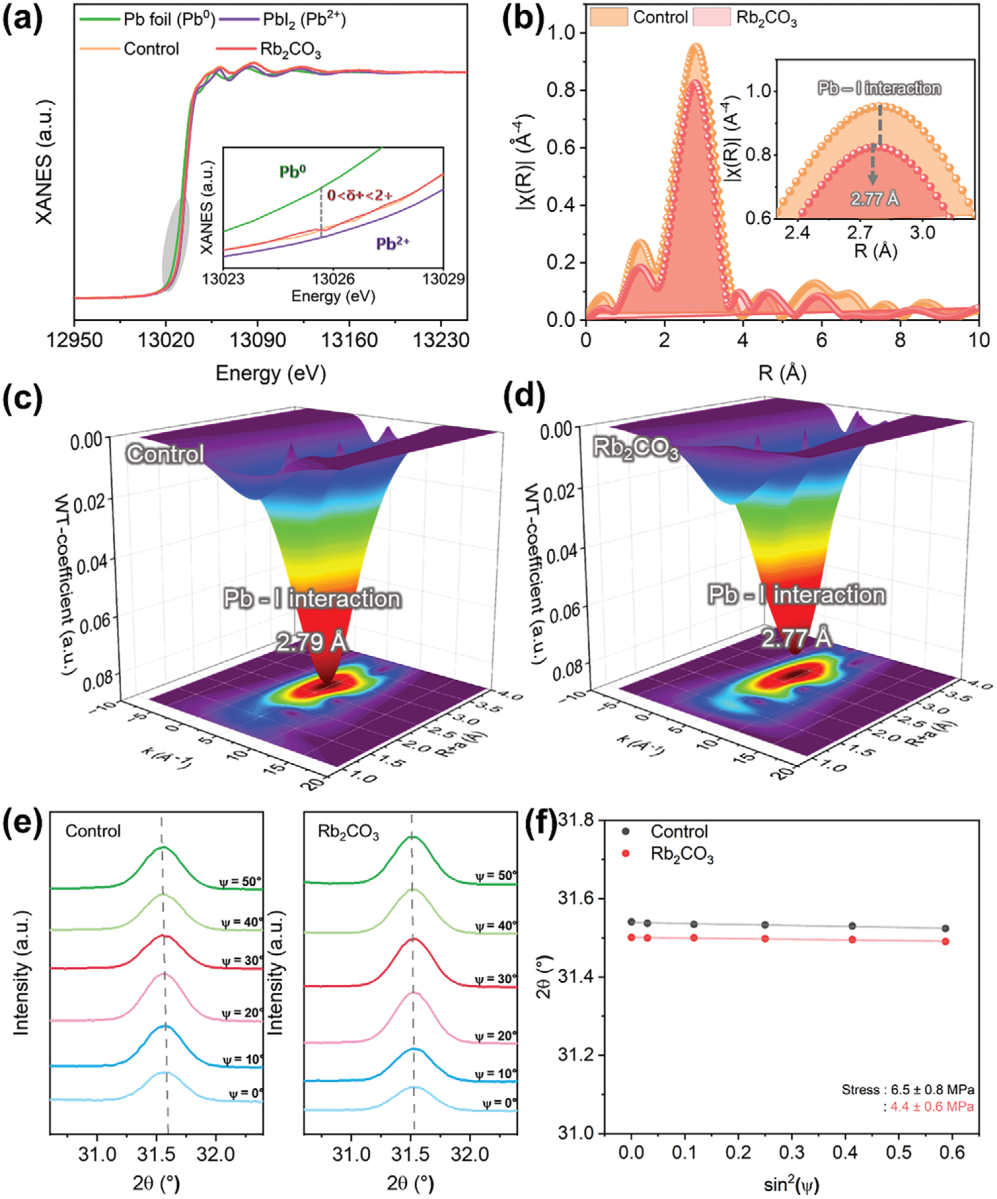
XAS measurements of perovskite films on SnO_2_ and SnO_2_/Rb_2_CO_3_ films a) Normalized Pb L_3_‐edged absorption coefficient. b) FT‐EXAFS spectra in *R*‐space for control and Rb_2_CO_3_‐treated perovskite films. c) Wavelet‐transformed EXAFS correlated with *k*‐space and *R*‐space for control, and d) Rb_2_CO_3_‐treated perovskite film. e) GIXRD spectra showing variations with different ψ values from 0° to 50° of control and Rb_2_CO_3_‐treated perovskite film. f) Linear fit of 2*θ*‐sin^2^(ψ) of control and Rb_2_CO_3_‐treated perovskite film.

Based on the aforementioned results, it was confirmed that Rb_2_CO_3_ with an optimal A‐cation effectively reduced interfacial defects and enhanced the crystallinity of the perovskite film. The optoelectronic properties of perovskites are profoundly affected by the optimized structure of corner‐sharing PbI_6_^2−^ octahedra, as they play a crucial role in mitigating the prevalent deep‐level trap states and SRH recombination centers associated with octahedral defects. To understand the effect of the Rb_2_CO_3_ treatment between SnO_2_ and perovskite on the local structure of perovskite films, XAS was conducted to reveal the atomic environment around Pb (II).^[^ ^47^ ^]^ **Figure** **4**a depicts the X‐ray absorption near‐edge structure, revealing a Pb L_3_‐edge absorption edge that hardly changed at ≈13 020 eV for the control and Rb_2_CO_3_‐treated perovskite films. This indicated that, even with Rb_2_CO_3_ treatment, the oxidation state of Pb^2+^ remained similar to that of PbI_2_, suggesting a stable chemical state of Pb^2+^.^[^ ^48^ ^]^ Furthermore, to prove the local chemical environments around Pb atoms precisely, *k^3^*‐weighted extended X‐ray absorption fine structure (EXAFS) with *k*‐space (Figure S31, Supporting Information) and the corresponding Fourier transformations of the EXAFS (FT‐EXAFS) spectrum (Figure 4b) were obtained. Specifically, fitted FT‐EXAFS in *R*‐space provided quantitative information on the coordination environments of the Pb–I bonds in the Rb_2_CO_3_‐treated film, encompassing the coordination number (CN), Debye–Waller (*D–W*) factors, and distance (*R*) (Figure S32, Supporting Information). The structural parameters obtained from the fitted data are listed in Table S4 (Supporting Information).^[^ ^49^ ^]^ Based on the fitted data, the *D–W* factor (σ^2^), indicating structural disorder of the perovskite film, decreased from 0.0213 ± 0.001 to 0.0167 ± 0.001 after Rb_2_CO_3_ interlayer insertion, implying a reduction in the distortion of the PbI_6_^2−^ octahedra induced by Rb_2_CO_3_ treatment. The first‐shell fitting of FT‐EXAFS exhibited a single intensity maximum attributed to the Pb–I bond, with a peak at 3.16 ± 0.015 Å for the control film and 3.15±0.015 Å for the Rb_2_CO_3_‐treated film (Figure 4b). This indicated the atomic dispersion and chemical stability of Pb coordinated with I atoms in the perovskite films and the shorter Pb–I bond distance indicated more robust and stable Pb–I bonding.^[^ ^50^ ^]^ The investigation of the CN of the PbI_6_^2−^ octahedra showed a higher CN of 6.54±0.327 in the control film compared to the ideal CN of 6, while upon Rb_2_CO_3_ treatment, the CN was reduced. The high CN value could be attributed to the formation of interstitial iodide (I_i_) or Pb–I antisite, the interaction of Pb atoms with additional coordination sites due to structural disorder or defects, or a substantial proportion of the *δ* phase adjacent to the *α* phase.^[^ ^51^ ^]^ The V_O_ at the SnO_2_/perovskite interface induced an increase in the Pb–I distance in the perovskite film, leading to the generation of I_i_ and facilitating PbI_6_^2−^ octahedra distortion, resulting in the *δ*‐FAPbI_3_ phase.^[^ ^13^ ^]^ However, passivated V_O_ in SnO_2_ by Rb_2_CO_3_ treatment suppressed PbI_6_^2−^ octahedra distortion and I_i_ formation, inhibiting the formation of *δ*‐FAPbI_3_ and PbI_2_. Additionally, the large thermal expansion coefficient of Rb_2_CO_3_ reduced the thermal expansion mismatch between the SnO_2_ substrate and perovskite layer, decreasing the residual stress and contracting the Pb–I bond distance in the octahedral structure, as indicated by the reduced structural parameters.^[^ ^52^ ^]^ The wavelet‐transformed EXAFS, illustrating the scattering pathways in both the *R*‐ and *k*‐spaces, is presented in Figure 4c,d. Additionally, factors from EXAFS for control and Rb_2_CO_3_‐treated perovskite films are presented in Figure S33 (Supporting Information).

Furthermore GIXRD using the 2*θ*‐sin^2^(ψ) method was employed to reconfirm the residual stress induced by Rb_2_CO_3_ treatment in the perovskite film.^[^ ^53^ ^]^ Figure 4e demonstrates a reduced peak shift of the (210) plane at 31.68° toward lower angles in the Rb_2_CO_3_‐treated film compared to the control film as the tilt angle ψ increases from 0° to 50°. The fitting result in Figure 4f, depicting 2*θ* as a function of sin^2^(ψ), indicates negative slopes for both the control and Rb_2_CO_3_‐treated films, suggesting the presence of tensile stress. Notably, the slope of the Rb_2_CO_3_‐treated film decreased from −0.02466 to −0.01654, corresponding to a decrease in residual stress from 6.5 ± 0.8 to 4.4 ± 0.8 MPa, indicating successful mitigation of residual stress by Rb_2_CO_3_ treatment.

Based on the XAS and GIXRD results, the Rb_2_CO_3_ interlayer between SnO_2_ and the perovskite alleviated the lattice distortion of the PbI_6_^2−^ octahedra and facilitated stress relaxation, confirming the formation of a stable corner‐sharing structure with passivated deep‐level trap states in the perovskite lattice.^[^ ^54^ ^]^ This showed good agreement with the reduced *E_U_* and improved TRPL results. Furthermore, the residual stress of other A_2_CO_3_ treatment can be confirmed in Figures S34 and S35, and Note S3 (Supporting Information).

**Figure 5:**
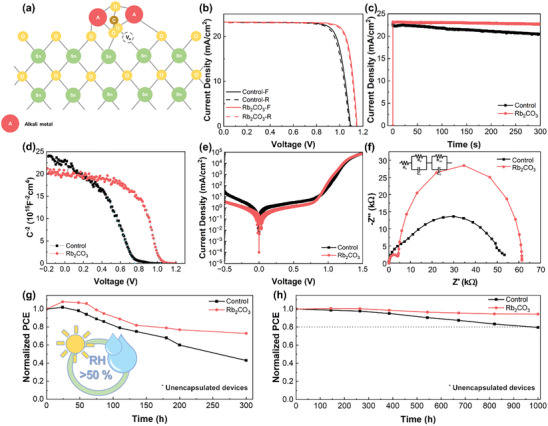
a) Schematic of the interaction between SnO_2_ layer and A_2_CO_3_ interlayer. b) *J–V* curves in reverse and forward scan directions for the control and Rb_2_CO_3_‐treated device. c) Steady‐state power output measured at a *V*_max_ of 0.822 V for the control and 0.932 V for the Rb_2_CO_3_‐treated device. d) Mott–Schottky plots, e) dark *J–V* curves, and f) Nyquist plots of PSCs based on SnO_2_ and SnO_2_/Rb_2_CO_3_ films. g) Stability performance of unencapsulated control and Rb_2_CO_3_‐treated PSCs at room temperature in ambient air (>50% RH) under continuous illumination for 300 h and h) at 20% RH and room temperature under dark conditions.

A schematic of the interaction between A_2_CO_3_ at the SnO_2_/perovskite interface was obtained (**Figure** **5**a). Based on the above characterization, it was established that A‐cations interact with O atoms on the SnO_2_ surface, and the two O atoms of CO_3_^2−^ establish single bonds with Sn atoms, thereby mitigating the formation of V_O_ in the SnO_2_ layer. At the SnO_2_/perovskite interface, increasing A‐cation size strengthens the binding with SnO_2_, reducing V_O_ formation, with Rb_2_CO_3_ identified as the optimal interfacial layer due to strong adsorption and minimal steric hindrance compared to Cs_2_CO_3_. Simultaneously, this interaction creates a dipole moment at the SnO_2_/perovskite interface, lowering the work function and achieving more favorable energy levels, thereby enhancing the carrier dynamics at the interface. A‐cations from A_2_CO_3_ may diffuse into the perovskite layer, inhibiting the recombination sites of the perovskite films and contributing to the suppression of native halide defect migration. Additionally, CO_3_^2−^ from A_2_CO_3_ exhibits a chemical affinity with undercoordinated Pb defects at the SnO_2_/perovskite interface, further enhancing the quality of both the bottom and top surfaces of the perovskite films. Consequently, Rb^+^, with its optimal ion size, not only effectively passivates the interfacial and internal defects of perovskite films but also leads to a reduction in residual stress within the perovskite film, contributing to improved structural stability and superior photovoltaic performance. Figure 5b illustrates the *J–V* hysteresis based on control‐ and Rb_2_CO_3_‐treated SnO_2_ films under forward and reverse scans, with the corresponding summary presented in Table S5 (Supporting Information). The efficiency improved from 20.11% to 22.10% with Rb_2_CO_3_ treatment. Additionally, the Rb_2_CO_3_‐treated devices displayed a weakened *J–V* hysteresis, with the hysteresis index (HI) decreasing from 0.021 to 0.013, as supported by the statistics presented in Figure S36 (Supporting Information), where the average HI value declined from 0.1046 to 0.0240. Hysteresis was mainly ascribed to the impediment of charge extraction at the interface resulting from excess ions caused by interfacial defects or defects in the bulk or surface of the perovskite layer.^[^ ^55^ ^]^ The effective mitigation of interfacial defects by Rb_2_CO_3_ and the efficient suppression of halide ion migration by Rb^+^ penetrating interstitial sites in the perovskite film contributed to a reduction in hysteresis. The investigation was then extended to measure the steady‐state power output under 1 sun illumination for 300 s at the maximum power point of the control device (0.822 V) and Rb_2_CO_3_‐treated PSCs (0.932 V), as shown in Figure 5c. These findings demonstrated that the device treated with Rb_2_CO_3_ exhibited superior and more consistently stable performance than the control device. Furthermore, the EQE spectra depicted in Figure S37 (Supporting Information) clearly show the impact of Rb_2_CO_3_ treatment, indicating a noticeable increase in the range from 440 to 780 nm compared to the control device.

To gain further insight into the enhanced charge‐carrier dynamics in PSCs, various electrical analyses were conducted. To explain the improvement in *V_OC_*, the built‐in potential (*V_bi_*) for devices was analyzed with and without Rb_2_CO_3_ treatment by measuring the Mott–Schottky plots (Figure 5d). The Rb_2_CO_3_‐treated device (*V_bi_* = 1.04 V) exhibited a higher *V_bi_* than the control (*V_bi_* = 0.76 V). This implied more effective separation and transport of the photo‐generated carriers, which led to a higher *J_SC_* and *V*_OC_. Figure 5e depicts the dark current for the control and Rb_2_CO_3_‐treated devices, with the Rb_2_CO_3_‐treated device exhibiting a retarded dark current density at low biases (−0.5 to 1.0 V) and a steep increase at biases exceeding 1.0 V. The lower dark current density at low biases indicated a reduced leakage current attributed to the passivated defects, whereas the increased current density at higher biases signified more efficient electron injection mobility in the device.^[^ ^56^ ^]^ Electrochemical impedance spectroscopy was then performed for the PSCs at 0.8 V under dark conditions to evaluate the charge recombination dynamics, and Nyquist plots were fitted with the inset equivalent circuit model (Figure 5f). Analysis of the parameters in Table S6 (Supporting Information) showed that the Rb_2_CO_3_‐treated PSCs exhibited a reduction in series resistance (*R_s_*) and charge transfer resistance (*R_tr_*), coupled with an enhancement in recombination resistance (*R_rec_*), compared to the control. This confirmed that Rb_2_CO_3_ treatment enhances the properties of charge extraction and transport at the SnO_2_/perovskite interface, mitigates charge recombination, and improves the *J_SC_* and *FF*.

The advancement of commercialized PSCs faces challenges owing to their instability, which largely stems from the susceptibility of perovskite films to moisture and light. To assess the long‐term stability of PSCs, unencapsulated control PSCs and those treated with Rb_2_CO_3_ were subjected to storage in ambient air with RH exceeding 50% while being continuously exposed to indoor light for 300 h. As shown in Figure 5g, both samples initially demonstrated improved efficiencies, but for the control device, efficiency sharply decreased to ≈43% of the initial efficiency after 300 h. In contrast, the Rb_2_CO_3_‐treated device exhibited high environmental stability, maintaining ≈73% of its initial efficiency under the same conditions for 300 h. Additionally, when examining the photos of perovskite films stored under identical conditions (Figure S38, Supporting Information), the film treated with Rb_2_CO_3_ did not show the presence of *δ*‐FAPbI_3_ even after 40 days, in contrast to the control film, indicating robust environmental stability. We further investigated the 1000 h stability of unencapsulated PSCs aged in ambient air environment with 20% RH under dark conditions, as shown in Figure 5h. In contrast to the control device, which exhibited a retention of ≈80% in the initial PCE, the Rb_2_CO_3_‐treated device demonstrated a sustained 94% of the initial PCE. The enhanced environmental stability could be assigned to the effective passivation of V_O_ at the interface, which led to a relaxed SnO_2_/perovskite interface and alleviated the lattice distortion of perovskite film.^[^ ^13^ ^]^ Additionally, Rb^+^ mitigated through ion diffusion and passivated the formation of unstable PbI_2_ and halide vacancy defects at the grain boundaries within the perovskite, thereby enhancing the intrinsic stability of the perovskite film.^[^ ^57^ ^]^

## 3 Conclusion

The effects of various alkali metal cations of alkali carbonate (Li_2_CO_3_, Na_2_CO_3_, K_2_CO_3_, Rb_2_CO_3_, and Cs_2_CO_3_) on the SnO_2_/perovskite interface were investigated. For the first time, the detailed role of alkali metal cations at the interface was elucidated and an optimal alkali metal cation was proposed for interfacial engineering. The size of alkali metal cations significantly influenced the V_O_ passivation capability of the SnO_2_ surface. Rb_2_CO_3_, with a moderate alkali ion size, forms strong absorption with SnO_2_, effectively passivating deep‐level defects and alleviating residual stress within perovskite films. Rb^+^ diffusion into the perovskite films further enhanced the structural stability of the perovskite. With a reduction in trap‐assisted non‐radiative recombination, Rb_2_CO_3_‐treated devices demonstrated an improved efficiency of 22.10% and an impressive *V_OC_* of 1.15 with improved environmental stability. This study provides insights into the crucial role of alkali metal cation selection in the efficient and stable design and optimization of perovskite solar cell interfaces.

## Conflict of Interest

The authors declare no conflict of interest.

## Author Contributions

W.J.J., E.H.K., and J.H.C. contributed equally to this work. D.L. and S.Y.K. contributed to supervision, project administration, and funding acquisition.

## Supporting information

Supporting Information

## Data Availability

The data that support the findings of this study are available from the corresponding author upon reasonable request.
