# Supplementary material for: Elucidating the Role of Alkali Metal Carbonates in Impact on Oxygen Vacancies for Efficient and Stable Perovskite Solar Cells
Source: Adv Sci (Weinh). 2024 Jul 25;11(36):2406657. doi: 10.1002/advs.202406657 (PMC11423148; doi:10.1002/advs.202406657)
Supplement: Supplementary file 1 — Supporting Information [file ADVS-11-2406657-s001.docx]

Supporting Information

***Elucidating the Role of Alkali Metal Carbonates in Impact on Oxygen Vacancies for Efficient and Stable Perovskite Solar Cells***

*Won Jin Jang^+,1^, Eun Ho Kim^+,2^, Jin Hyuk Cho^+,1^, Donghwa Lee*^,2^, Soo Young Kim^*,1^*

^1^Department of Materials Science and Engineering Korea University, Seoul 02841, Republic of Korea

^2^Department of Material Science & Engineering, Pohang University of Science and Technology (POSTECH), Pohang, 37673 South Korea

*Corresponding Author

Prof. D. H. Lee; E-mail: donghwa96@postech.ac.kr

Prof. S. Y. Kim; E-mail: sooyoungkim@korea.ac.kr

*^+^* These authors contributed equally to this work

**Experimental Section**

**Materials**

Lead(II) iodide (PbI_2_, 99.99%) and 2,2′,7,7′-Tetrakis-(*N*,*N*-*di*-4-methoxyphenylamino)-9, 9′-spirobifluorene (Spiro-MeOTAD) were purchased from Tokyo Chemical Industry and Luminescence Technology, respectively. Formamidinium iodide (FAI, >99.99%), methylammonium chloride (MACl, >99.99%), and FK 209 Co(III) TFSI salts were purchased from Greatcell Solar Materials. Ethanol, 2-propanol, was purchased from Daejung, and tin(II) chloride dihydrate was purchased from Alfa Aesar. [6,6]-Phenyl C61 butyric acid methyl ester was purchased from OSM. Unless otherwise stated, all materials were purchased from Sigma-Aldrich.

**Device Fabrication**

Fluorine-doped tin oxide (FTO) glass (Ashai Glass) was cleaned using sequential sonication in deionized water, 2-propanol, and acetone for 15 min each. The cleaned FTO glass was then dried in nitrogen and subjected to UV-ozone treatment for 10 min. The SnCl_2_ precursor (0.16 M SnCl_2_·2H_2_O) was diluted in ethanol for the deposition of the SnO_2_ layer. The SnCl_2_ precursor was spin-coated onto the cleaned FTO substrate at 4000 rpm for 30 s and then annealed at 180 °C for 1 h. After cooling to room temperature, the film was subjected to UV-ozone treatment for 10 min. To prepare the A_2_CO_3_ precursor, A_2_CO_3_ (0.3, 0.5, 0.7, 1.0 mg/ml) was dissolved in deionized water and stirred for at least 2 h to obtain a clear solution. The A_2_CO_3_ precursor was spin-coated onto the SnO_2_ substrate at 4000 rpm for 30 s and annealed at 150 °C for 20 min. To prepare the perovskite precursor, FAI (274 mg), PbI­_2_ (734 mg), MACl (36 mg), and methylammonium lead tribromide (MAPbBr_3_; 40 mg) were dissolved in a mixed solvent (anhydrous N,N-dimethylformamide/dimethyl sulfoxide; 8:1 v/v). The (FAPbI_3_)_0.95_(MAPbBr_3_)_0.05_ solution was spin-coated on a SnO_2_/A_2_CO_3_ substrate at 4000 rpm for 30 s, and diethyl ether quickly dropped onto the substrate after 10 s during the spinning procedure. The resulting yellowish adduct film was annealed at 150 °C for 15 min to form a photoactive perovskite film. A solution of spiro-OMeTAD was prepared, comprising spiro-OMeTAD (45 mg), 4-*tert*-butyl pyridine (19.6 μL), a lithium bis(trifluoromethanesulfonyl)imide (Li-TFSI) solution (11.5 μL; 517 mg of Li-TSFI in 1 mL of acetonitrile), and a FK 209 Co(III) TFSI solution (5 μL; 376 mg of Co-TFSI in 1 mL of acetonitrile) in chlorobenzene (0.5 mL). This solution was spin-coated at 2000 rpm for 30 s. Subsequently, a 90-nm-thick Ag electrode was deposited through a shadow mask using thermal evaporation at a constant evaporation rate of 1.3 Å s^−1^.

**Characterization**

The current density–voltage (*J–V*) characteristics of the perovskite solar cells (PSCs) were determined using a Keithley 2614 B source meter under AM 1.5 G illumination (Oriel 150 W solar simulator) and ambient conditions. Both forward (−0.1 to 1.2 V) and reverse scans (1.2 to −0.1 V) were performed, and the active area of the device was 0.14 cm^2^ without anti-reflection coating. The external quantum efficiency (EQE) was ayalyzed by a Newport-Oriel IQE200. X-ray photoelectron spectroscopy (XPS) was conducted using K*α*+ (ThermoFisher Scientific) measurement with an Al *Kα* micro-focused monochromator, and all XPS spectra were calibrated using C 1s (284.8 eV). X-ray diffraction (XRD) patterns were acquired using a D8-Advance/Bruker-AXS device with monochromatized Cu *Kα* radiation (λ = 1.5418 Å). The morphologies and elemental distributions of the perovskite and SnO_2_ films were investigated using field emission-scanning electron microscopy (FE-SEM; SIGMA, Carl Zeiss) and energy-dispersive X-ray spectrometry (EDS; Thermo NORAN System 7). The optical properties of the films were analyzed by Ultraviolet-visible (UV-vis) spectroscopy (FP-8550, JASCO) in the wavelength range 500–850 nm. The roughness and energy states of the SnO_2_ films were characterized using atomic force microscopy (AFM; XE-100, Psia) and ultraviolet photoelectron spectroscopy (UPS; Thetaprobe base system and Nexsa XPS system, Thermo Fisher Scientific). The water contact angles of the SnO_2_ films were determined using a contact angle goniometer (Phoenix 150, SEO, South Korea). Fourier transform infrared (FTIR) spectra was performed in a attenuated total reflection (ATR) mode in the rage of 400 and 4000 cm^-1^ by NICOLET iS50 FTIR spectrometer.

Time-resolved photoluminescence (TRPL) was performed using a confocal microscope (MicroTime-200, PicoQuant, Germany). Lifetime measurements were performed at the Korea Basic Science Institute (KBSI), Daegu Center, Korea. A single-mode pulsed diode laser (470 nm with a pulse width of ~30 ps and an average power of ~50/~200 nW in 0.5/2 MHz laser repetition) was used as the excitation source. A dichroic mirror (490 DCXR, AHF), a 100-μm pinhole, a longpass filter (FEL0700, Thorlabs), and a single photon avalanche diode (PDM series, MPD) were used to collect emission from the samples. A 10× (air) objective was used to illuminate the film samples. A time-correlated single-photon counting system (PicoHarp300, PicoQuant GmbH, Germany) was used to count the emitted photons. Photoluminescence (PL) lifetime images consisting of 200 × 200 pixels were recorded using a time-tagged time-resolved (TTTR) data acquisition method. Exponential function fitting for the obtained emission decays was performed using SymphoTime 64 software (ver. 2.2). Steady-state PL spectra were measured by guiding the emission photons through an optical fiber to an external spectrophotometer (F-7000, Hitachi).

Time-of-flight secondary ion mass spectroscopy (TOF-SIMS) experiments were performed using a TOF-SIMS 5 (ION-TOF GmbH, Münster, Germany) at the KBSI Busan Center using a pulsed 30-keV Bi_3_^+^ primary beam with a current 0.33 pA. The analyzed area used in this work is a square of 200 μm × 200 μm. Positive ion spectra were internally calibrated using H^+^, CH_3_^+^, C_2_H_5_^+^, C_3_H_7_^+^, and C_4_H_9_^+^, and negative ion spectra were internally calibrated using H^–^, C^–^, C_2_^–^, C_3_^–^, and C_4_^–^ peaks normalized to the respective secondary total ion yields. Chemical images of the analyzed area were recorded at 128 × 128 pixel resolution during data acquisition. The sputtered area was a square of 500 μm × 500 μm obtained using a 10-keV Ar cluster for depth profile.

X-ray absorption spectroscopy (XAS) was performed in transmittance and fluorescence detection modes at beamlines 7D and 8C at the Pohang Accelerator Laboratory. The XAS data, including the X–ray absorption near–edge structure (XANES) and extended X-ray absorption fine structure (EXAFS) data, were analyzed using the ATHENA and ARTEMIS programs of the IFEFFIT package based on the standard XAS equation. The WT-EXFAS technique utilizes the modulus of wavelet-transformed coefficients to simultaneously examine the absolute value coefficients in both *k*-space and *r*-space.

Grazing incidence X-ray diffraction (GIXRD) was conducted using high resultion x-ray diffractometer (PANalytical, Empyrean) at the KBSI Daegu Center. The residual stresses ($\sigma$) were determined using the equation: $\sigma= -\frac{E}{2(1+\nu)}\frac{\pi}{180}cot\theta_{0}\frac{\partial(2\theta)}{\partial{sin}^{2}(\psi)}$, where *E* is the Young’s modulus, *ν* is the Poisson’s ration, and *θ_0_* is the Bragg angle. *E* and *ν* were calculated using values of 10 GPa and 0.3, respectively.^[1,2]^

Space-charge-limited current, steady-state power output, Mott–Schottky plot analysis, electrochemical impedance spectroscopy, and dark current-voltage (*I–V*) curves were obtained using an Ivium V55630 potentiostat.

**DFT calculations**

All DFT calculations presented in this study were conducted using the Vienna Ab initio Simulation Package.^[3]^ Electron-ion interactions were described using the plane-wave projector augmented wave method,^[4]^ and the Perdew–Burke–Ernzerhof function was utilized for exchange-correlation calculations.^[5]^ Dispersion interactions were corrected using the DFT-D3 method proposed by Grimme.^[6]^ A plane-wave cutoff energy of 520 eV was applied across all calculations. The rutile structure of SnO_2_ with space group P4_2_/mnm was employed, and the optimized lattice constants agreed with the experimental parameters.^[7]^ A 3 × 2 supercell with a slab thickness of six layers was constructed for this study. The Brillouin zone was sampled using a 4 × 3 × 1 Monkhorst-pack k-point grid. Full relaxation of all the structures was performed until the energy and forces met the convergence criteria of 10^–6^ eV for energy and 0.02 eV/ Å for atomic forces. A vacuum thickness of 20 Å was implemented to prevent interactions between periodic slabs. The bottom two layers were constrained to remain at the equilibrium position of the bulk, whereas the bottom layer was passivated with hydrogen atoms to mitigate unphysical charge-transfer effects. The defect formation energy (*DFE*) of oxygen vacancy (V_O_) is computed as follows:

*DFE*(V_O_) = *E_tot_*(SnO_2_–V_O_) – *E_tot_*(SnO_2_) + *μ_O_*

*μ_O_* = 0.5∙*E_tot_*(O_2_)

where *DFE*(V_O_) is *DFE* of V_O_, *E*(SnO_2_–V_O_) is the total energy of V_O_ contained system, *E*(SnO_2_) is the total energy of a perfect system, *E*(O_2_) is the total energy of oxygen gas, and *μ_O_* is the chemical potential of oxygen. The *DFE* of V_O_ on the A_2_CO_3_-adsorbed surface was investigated by removing O from the surface.


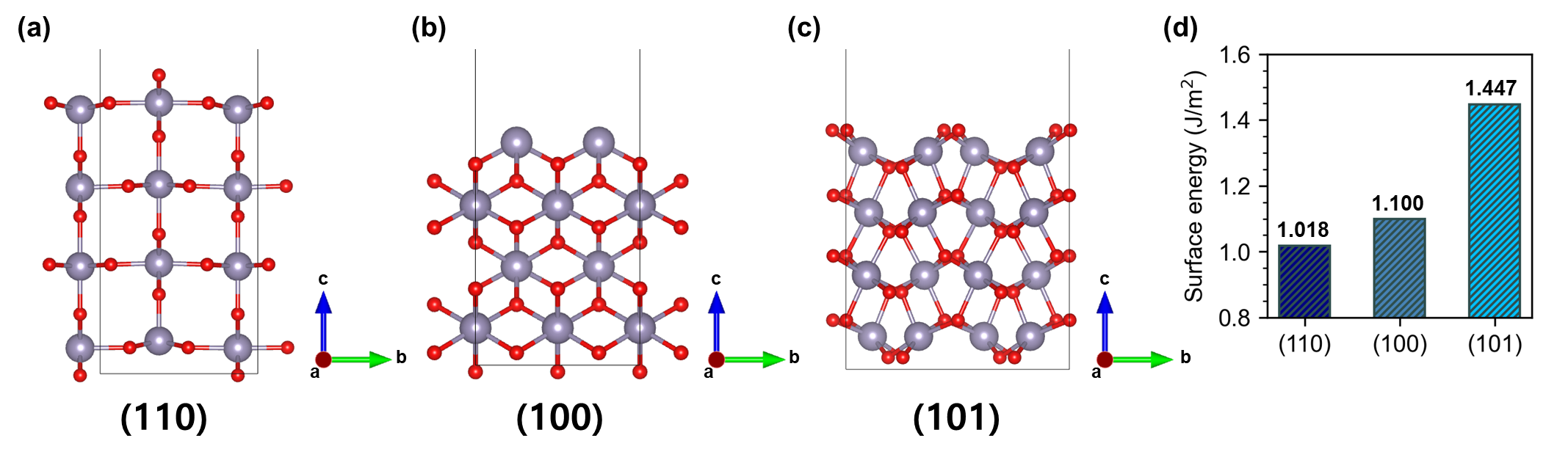


**Figure S1.** (a)–(c) Depiction of the optimized structures for SnO_2_ (110), (100), and (101) slab systems. (d) Plot of the corresponding surface energy, with (110) exhibiting the lowest surface energy among the three surfaces.


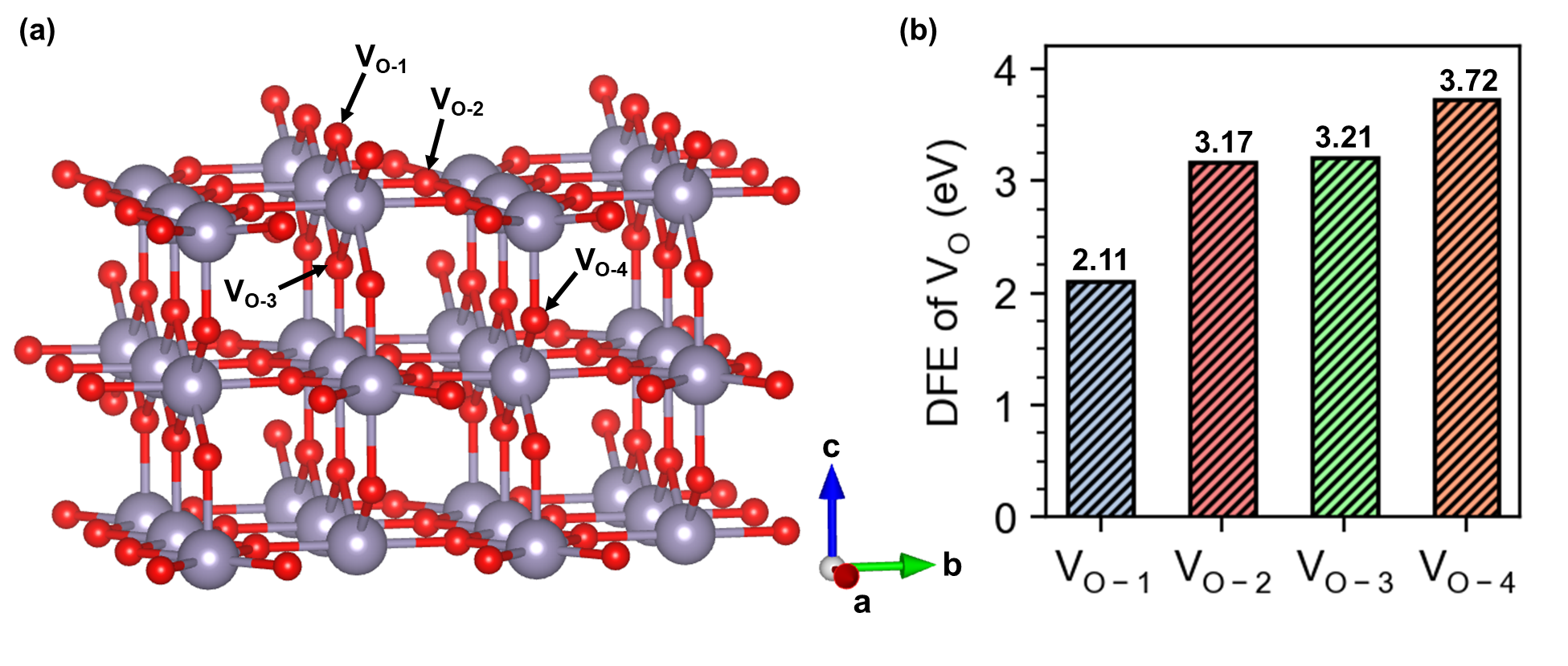


**Figure S2.** (a) Identification of different V_O_ sites on the SnO_2_ (110) surface. (b) Plot of the calculated defect formation energy (*DFE*) of V_O_ for each respective site. The bridging site on the top layer (V_O–1_) is identified as the most energetically stable defect, exhibiting a *DFE* of V_O_ that is 1.06 eV lower than that of the subsurface site (V_O–3_).

**
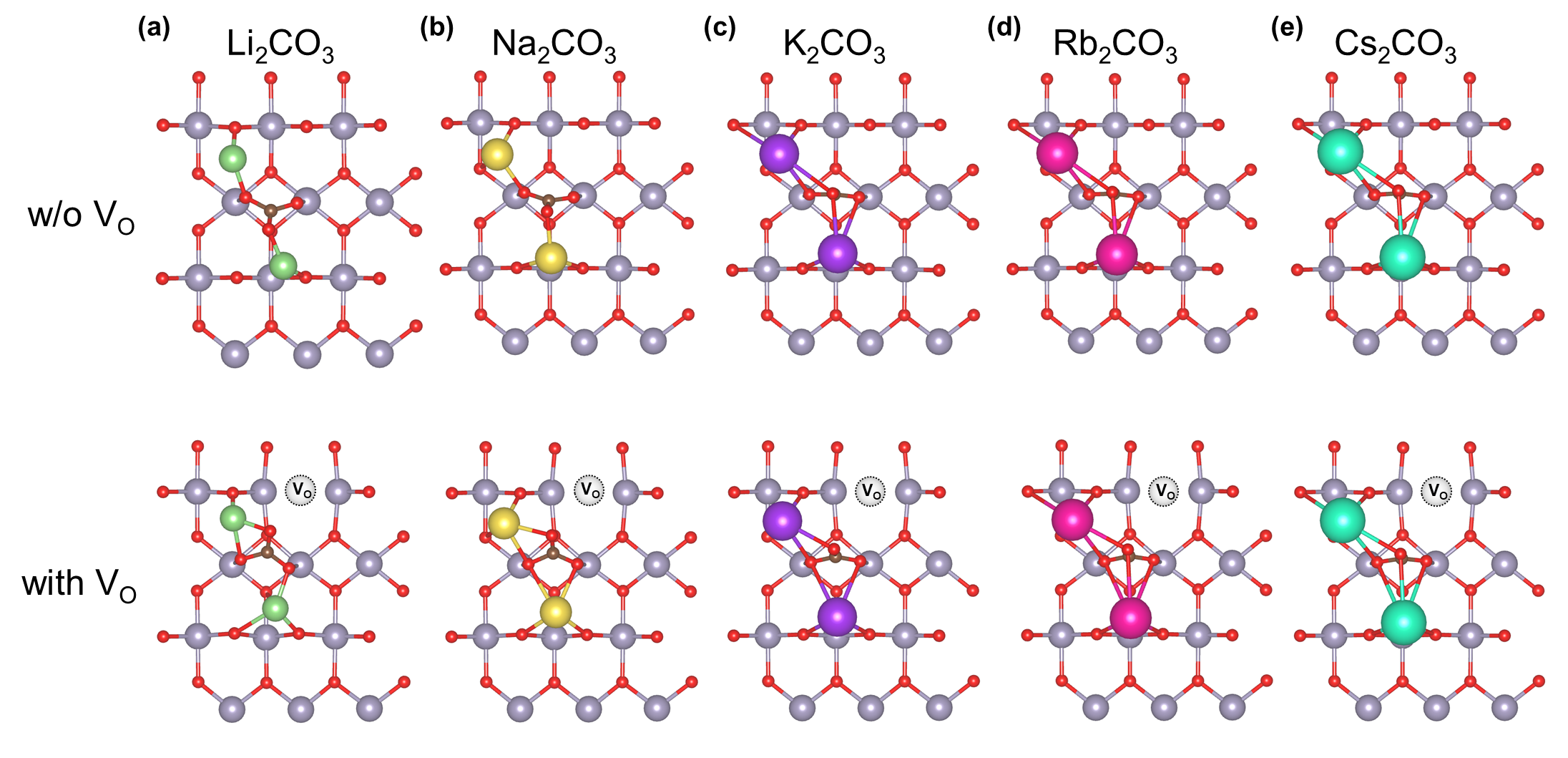
**

**Figure S3.** Upper figure displays the top view of the optimized structure of the SnO_2_–A_2_CO_3_ system for (a) Li_2_CO_3_, (b) Na_2_CO_3_, (c) K_2_CO_3_, (d) Rb_2_CO_3_, and (e) Cs_2_CO_3_ without an oxygen vacancy (V_O_). The lower figure showcases the same top view but with the inclusion of V_O_ in the system. The illustrations exclusively depict the top layer of the surface.

**
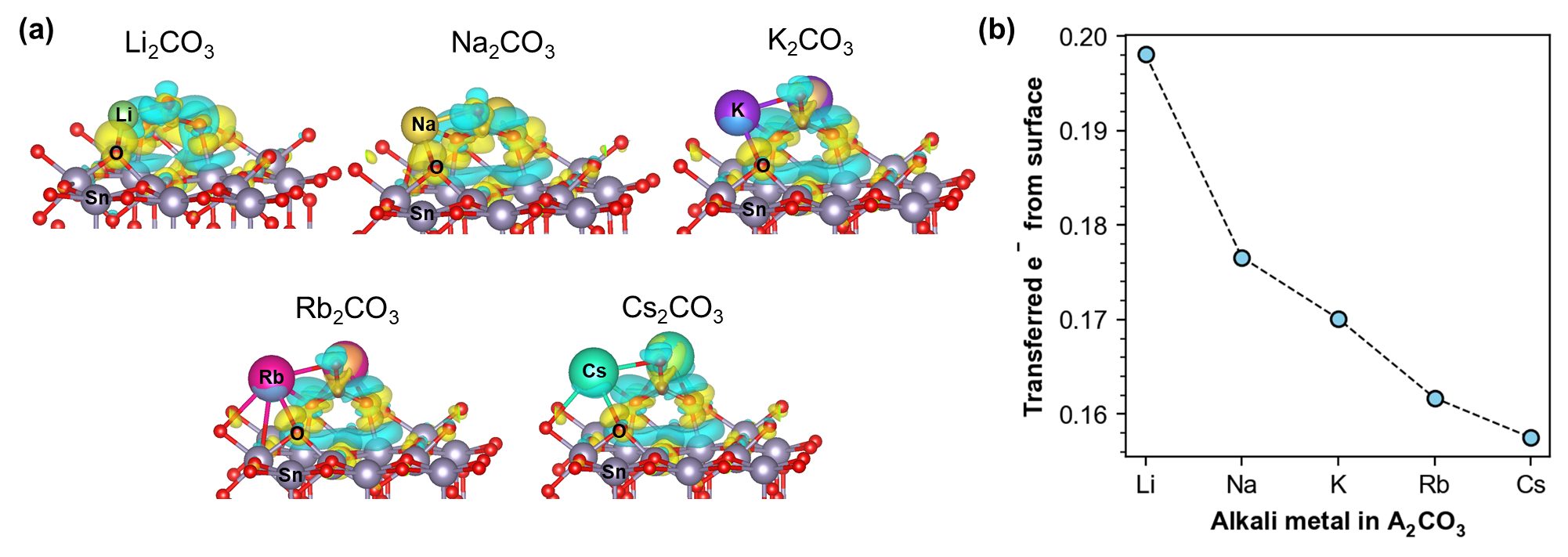
**

**Figure S4.** (a) Charge density difference (Δ*ρ*) plots illustrating the interaction between A_2_CO_3_ (A=Li, Na, K, Rb, and Cs) and the SnO_2_ surface with an V_O_ (SnO_2_–V_O_). Yellow and blue colors signify the gain and loss of electrons, respectively. (b) Bader charge analysis of transferred charge from SnO_2_–V_O_ to A_2_CO_3_. Reduced charge transfer from surface to A_2_CO_3_ from Li to Cs explains observed upward trend of *DFE* of V_O_.





**Figure S5.** XPS spectra of a survey scan of a SnO_2_ film.


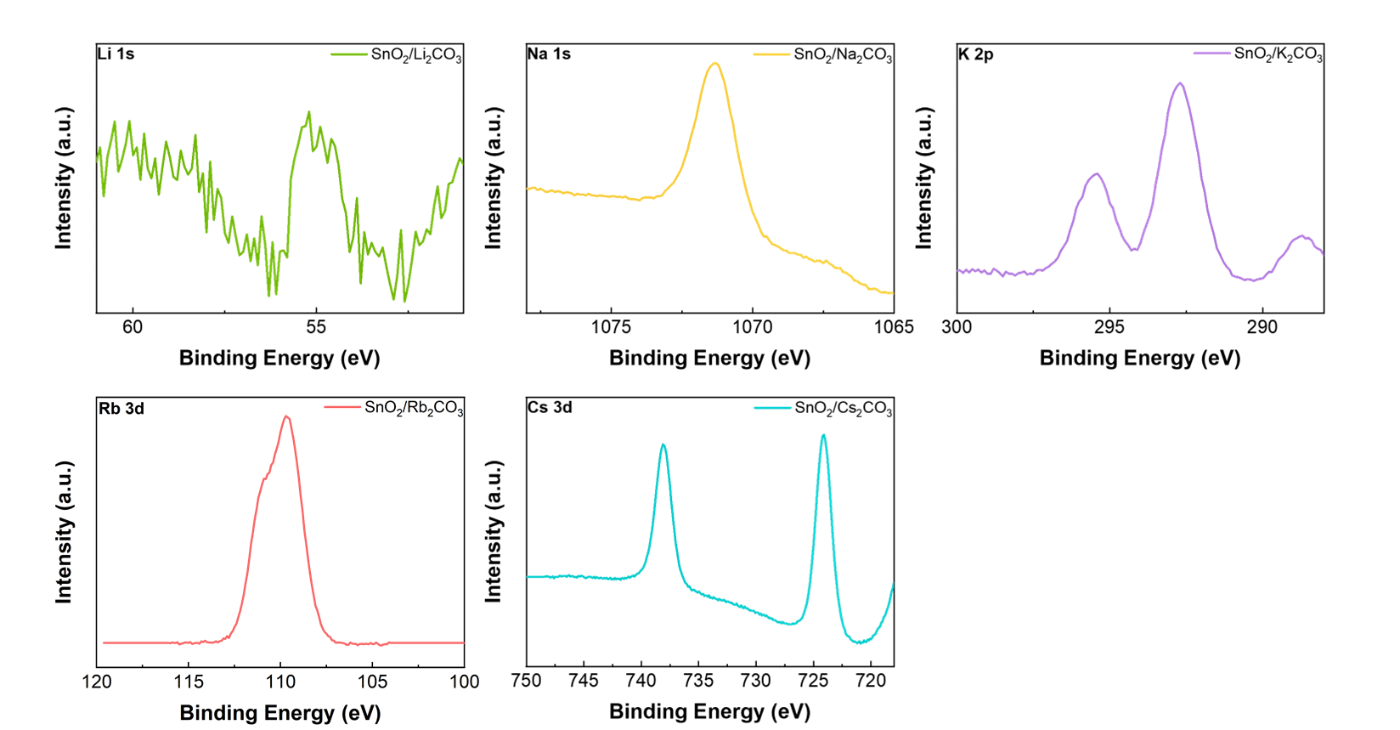


**Figure S6.** XPS spectra of A_2_CO_3_-treated SnO_2_ films for A-cations peaks.

**
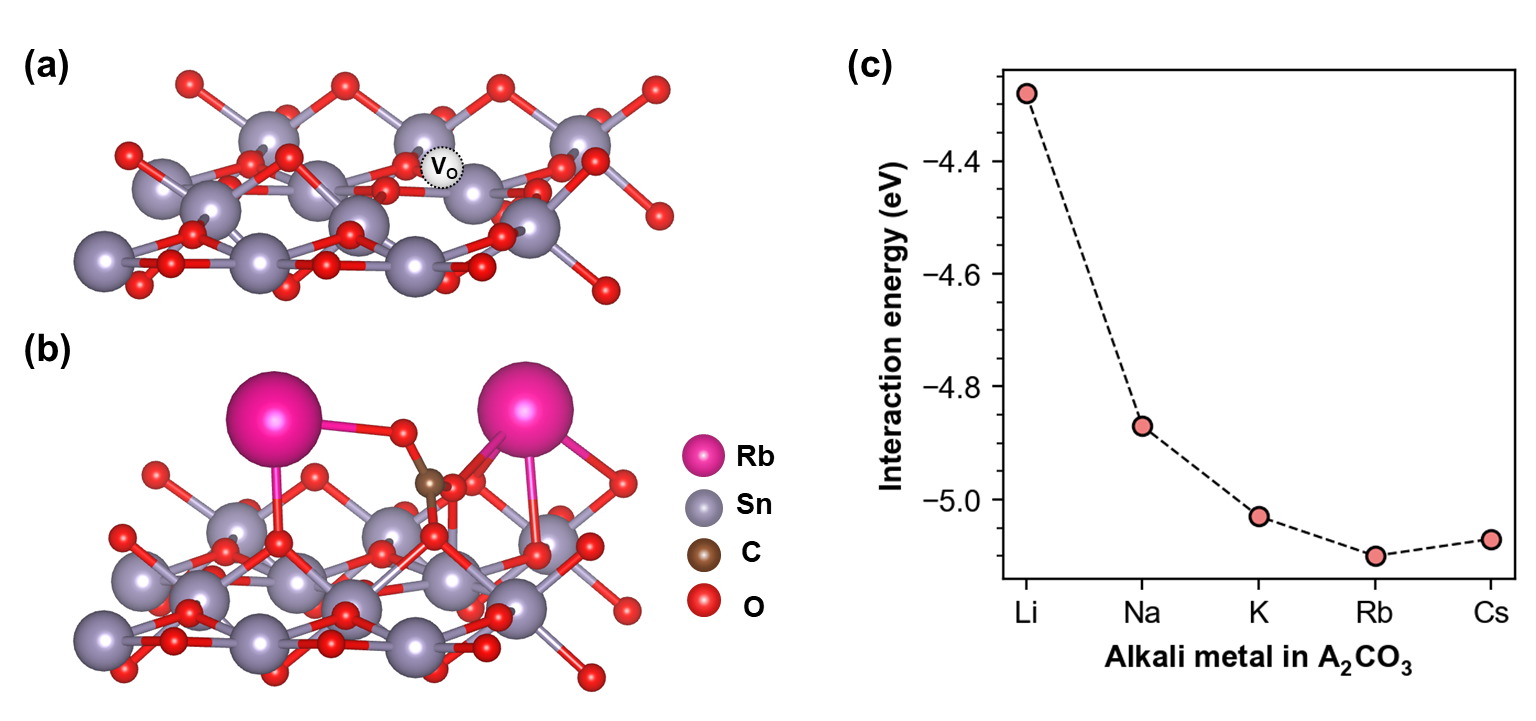
**

**Figure S7.** (a) Schematic view of top layer in V_O_-contained SnO_2_ surface (SnO_2_–V_O_). (b) V_O_ is passivated with O in Rb_2_CO_3_. (c) Evaluation of the interaction energy between V_O_ and A_2_CO_3_ (A=Li, Na, K, Rb, and Cs). Interaction energy is calculated as the energy difference before (A_2_CO_3_ in vacuum) and after passivation of V_O_. The lowest interaction energy is observed in Rb_2_CO_3_.


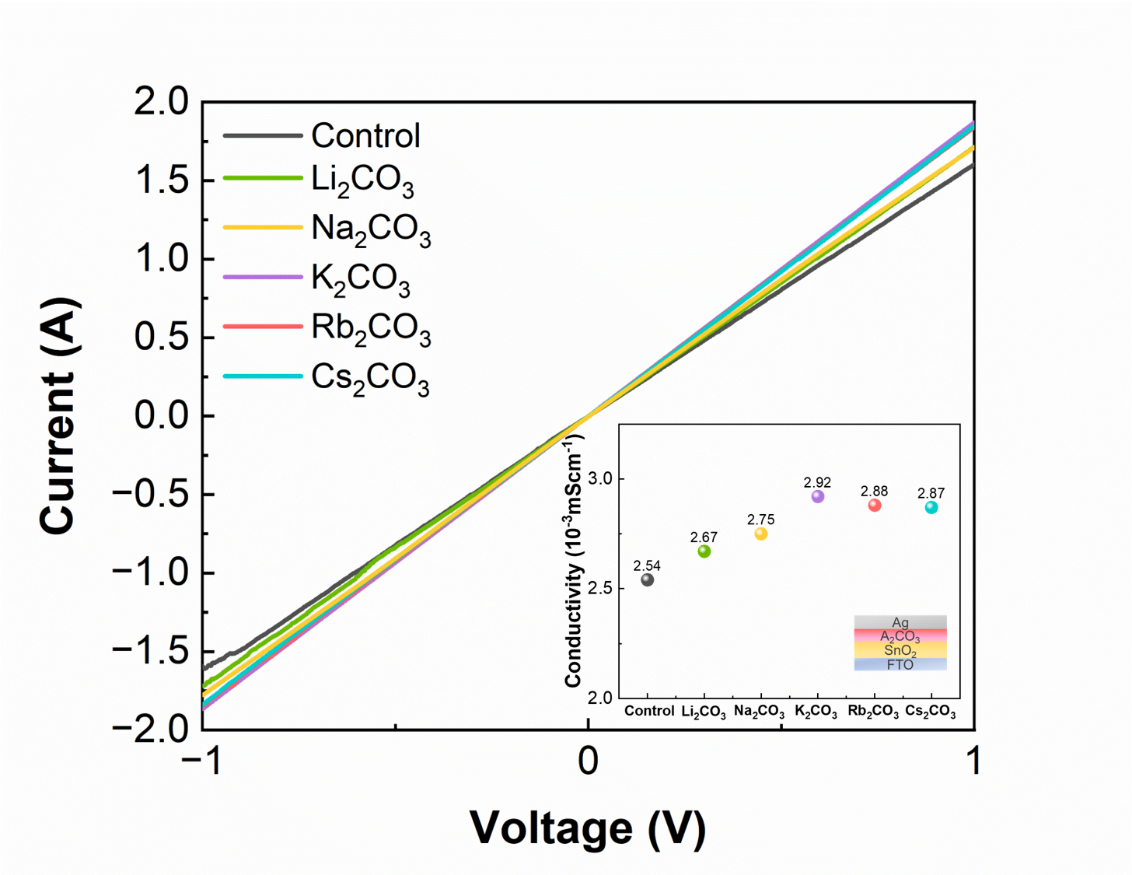


**Figure S8.** *I–V* curves of FTO/SnO_2_/Ag and FTO/SnO_2_/A_2_CO_3_/Ag devices. Conductivity (σ) is determined using $\sigma=\frac{Id}{VA}$ (where d and A represent the thickness and area of the SnO_2_ films, respectively). (The inset figure is the calculated conductivity of pristine and A_2_CO_3_-treated SnO_2_ films in the dark.)


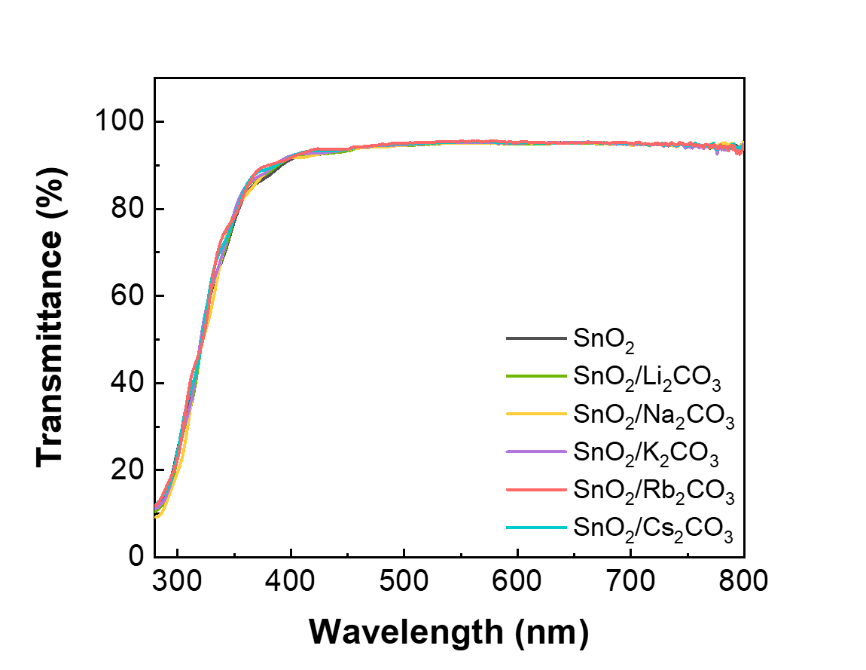


**Figure S9.** Transmittance spectra of SnO_2_ and SnO_2_/A_2_CO_3_ films.





**Figure S10.** Intensity ratio of the PbI_2_ peak to the (100) peak of perovskite film from XRD patterns. The significant decrease in the PbI_2_ peak to the (100) peak ratio from 0.267 to 0.134 for Rb_2_CO_3_ treatment, compared to that of the control film, could be attributed to the suppressive capability of Rb^+^ on the growth of the *δ*-phase FAPbI_3_ and PbI_2_.





**Figure 11**. The reciprocal of full width half-maximum (FWHM) of (100) peak in perovskite films with and without A_2_CO_3_ treatment analyzed using XRD spectra.


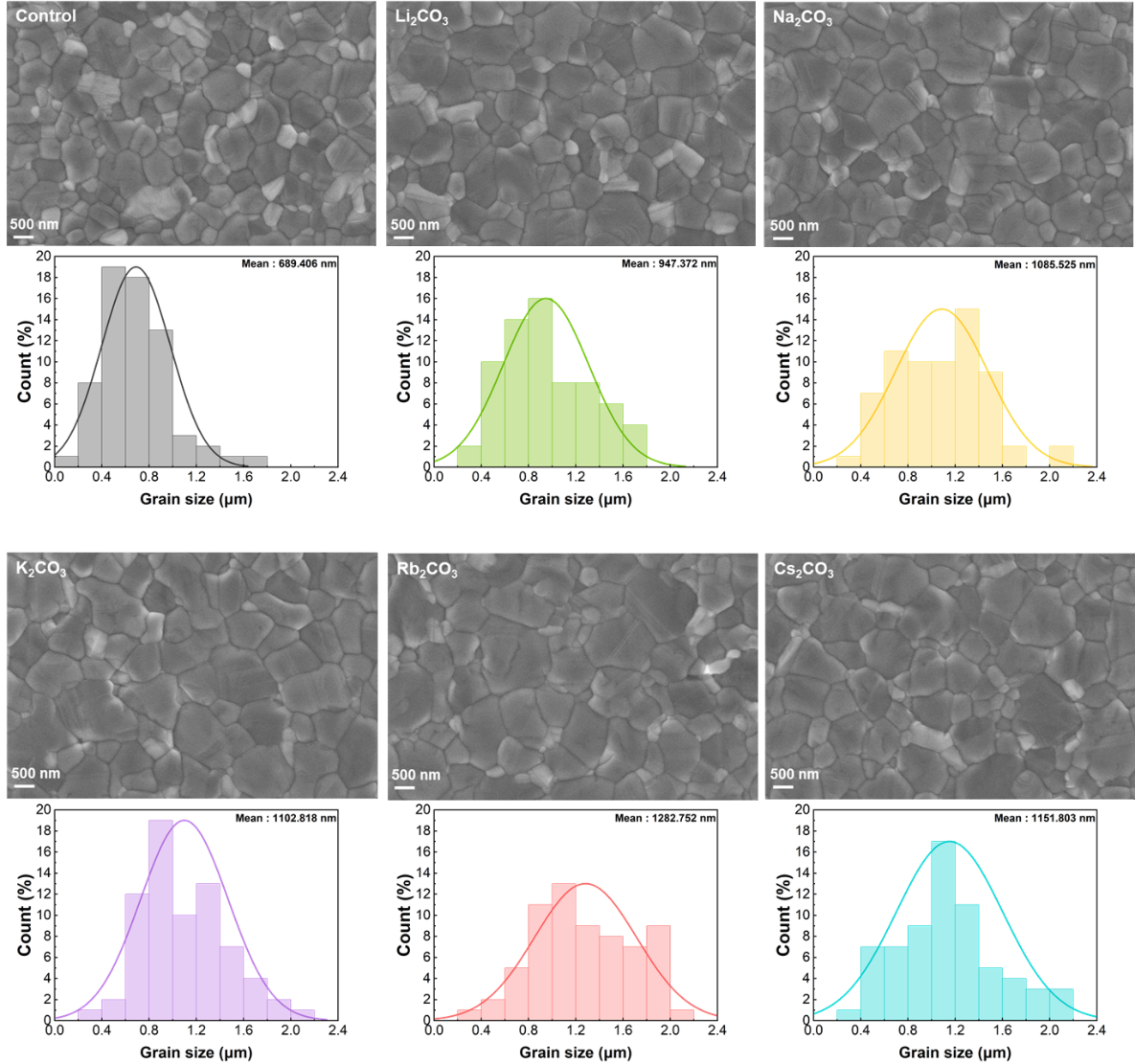


**Figure S12.** FE-SEM images and grain size distribution of perovskite and A_2_CO_3_/perovskite films deposited on SnO_2_ films.





**Figure S13.** UV-vis absorption spectra and A_2_CO_3_/perovskite films deposited on SnO_2_ films.


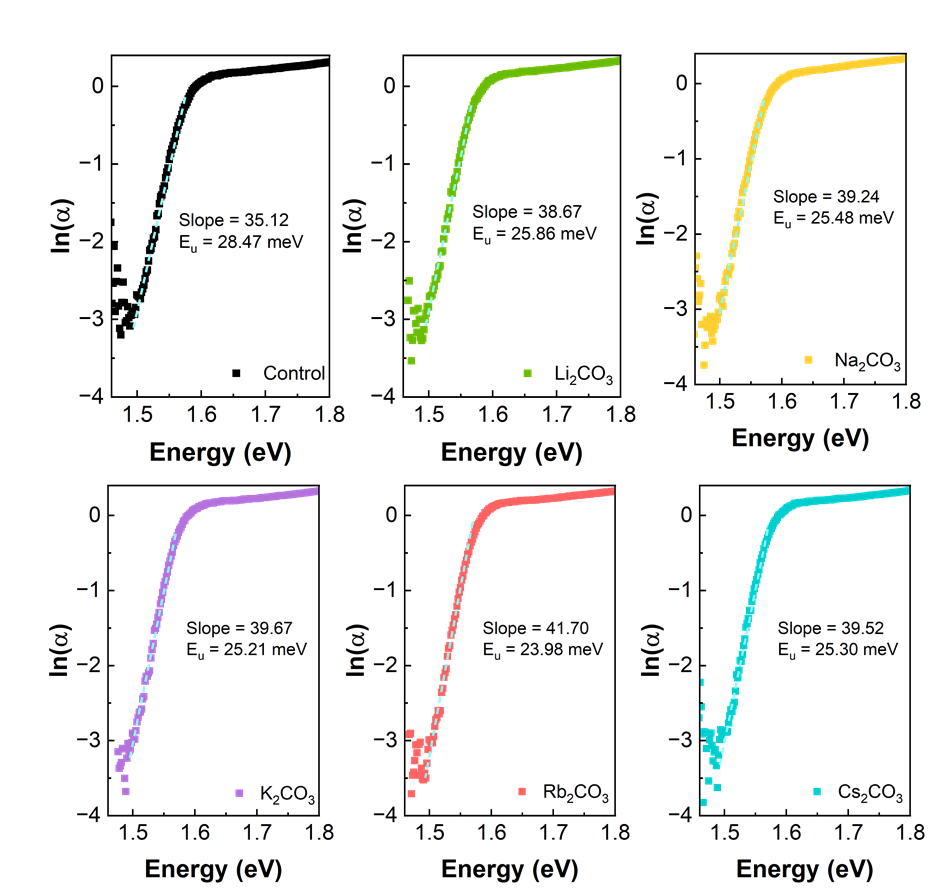


**Figure S14.** Urbach energy of control and A_2_CO_3_-treated perovskite films. (FTO/SnO_2_/A_2_CO_3_/perovskite) The decrease in *E_U_* indicates improved crystallization of perovskite, suggesting that A_2_CO_3_ treatment induces reduced structural disorders and a lower electron trap state density.

**Note S1:** The presence of lattice vibrations and defects in the absorption spectra of perovskites results in Urbach tails that decay exponentially below the bandgap. The steepness of these Urbach tails allows for the determination of the Urbach energy.^[8]^

$$\alpha=\alpha_{0}exp(\frac{hv}{E_{U}})$$

where *E_U_* is the Urbach energy, $\alpha$_0_ is the absorption constant, and $\alpha$ is the absorption coefficient. Urbach energy, indicated by the width of the Urbach tail, offers valuable insights into the structural defects and disorders within the lattice structure of perovskites, making it a significant physical characteristic. Consequently, perovskite films with high Urbach energies lead to increased structural disorders and trap-assisted recombination. Specially, *E_U_* is associated with deep-level defect levels that induce Shockley–Read–Hall recombination, demonstrating a strong correlation with open-circuit voltage (*V_OC_*)*_._*^[9]^


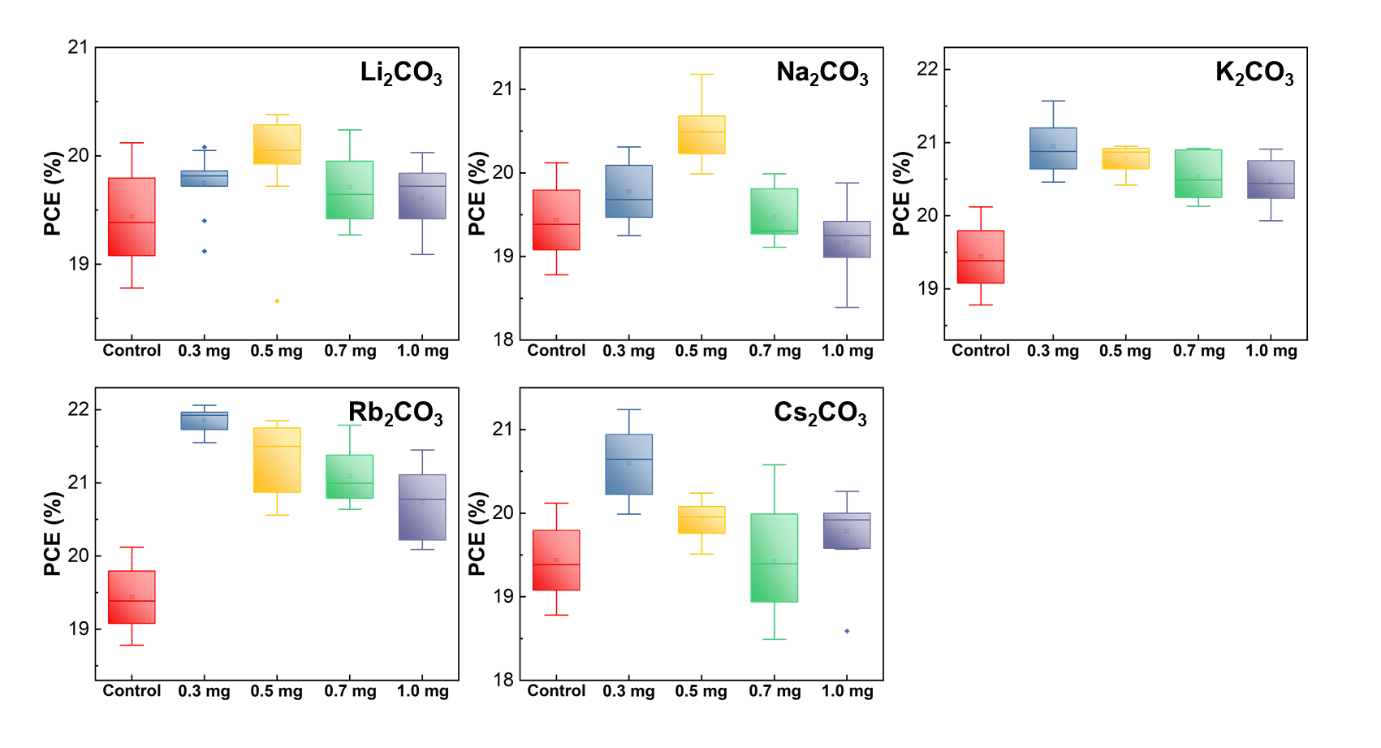


**Figure S15.** Photovoltaic parameters of devices with control SnO_2_ and A_2_CO_3_-treated SnO_2_ films with different concentrations.


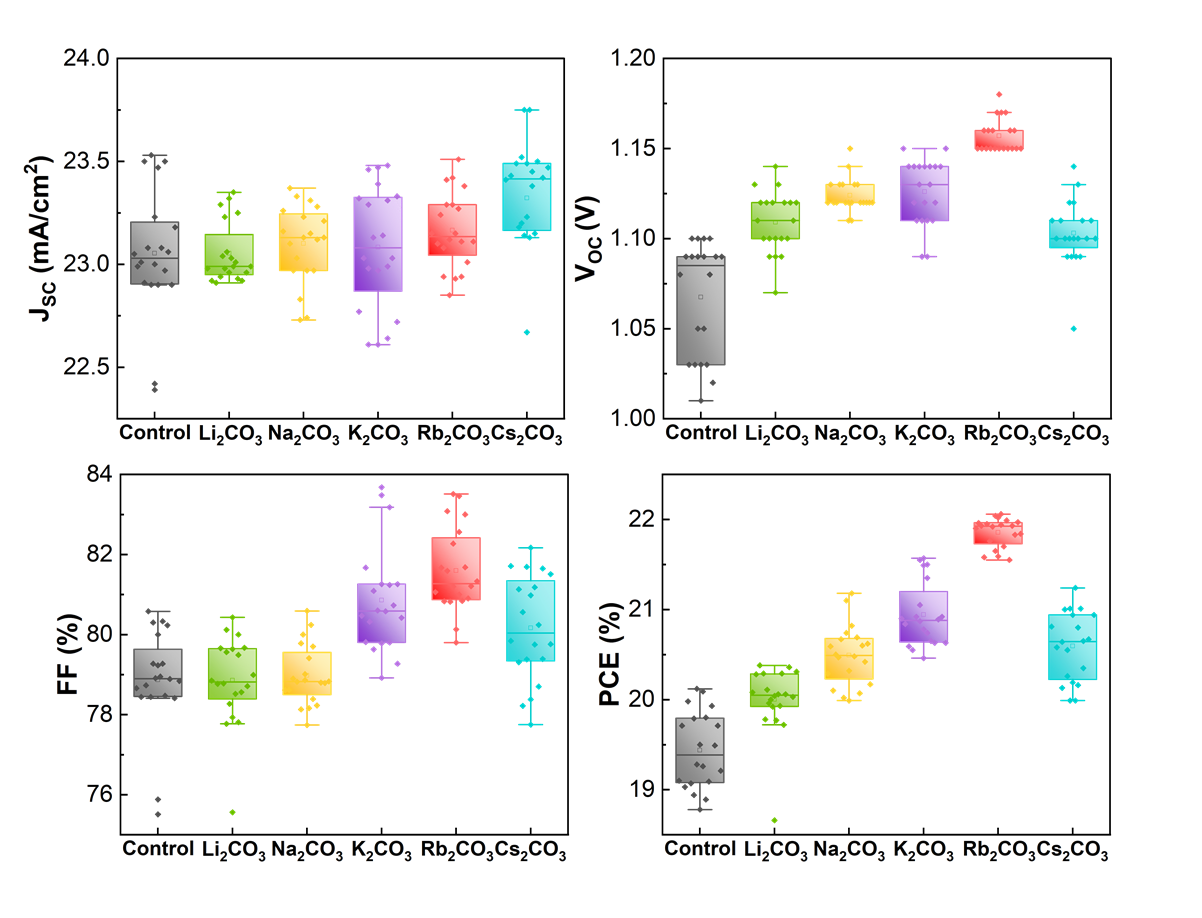


**Figure S16.** Statistical diagrams of photovoltaic parameters of devices with control SnO_2_ and A_2_CO_3_-treated SnO_2_ films for 20 devices.

**

**

**Figure S17.** EQE spectra and integrated short-circuit current density (*J_SC_*) of PSCs with and without A_2_CO_3_ treatment. The integrated *J_SC_* values derived from EQE spectra were 23.45 mA/cm² for control device, 22.83 mA/cm² for Li_2_CO_3_-treated device, 23.45 mA/cm² for Na_2_CO_3_-treated device, 23.83 mA/cm² for K_2_CO_3_-treated device, 24.16 mA/cm² for Rb_2_CO_3_-treated device, and 23.97 mA/cm² for Cs_2_CO_3_-treated device.


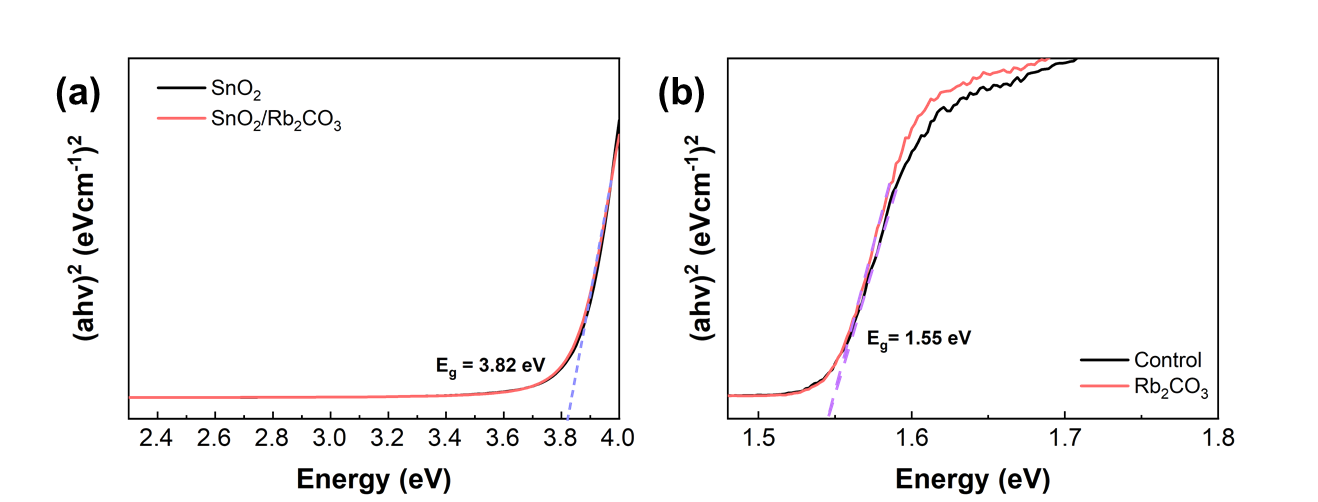


**Figure S18.** Tauc plot of (a) SnO_2_ and SnO_2_/Rb_2_CO_3_ films, and (b) perovskite and Rb_2_CO_3_/perovskite film.


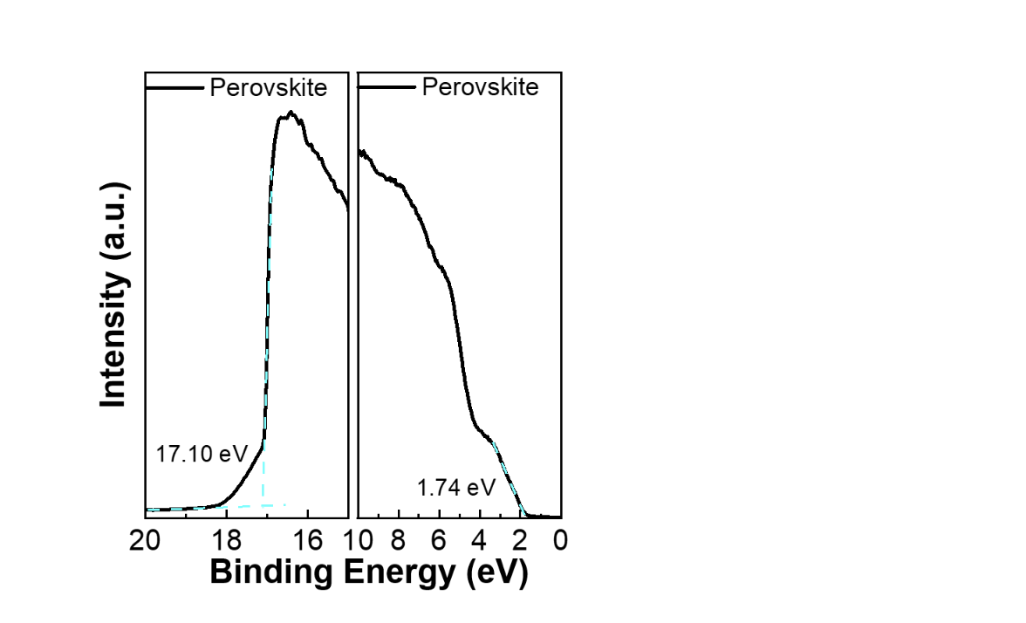


**Figure S19.** UPS spectra of perovskite film.


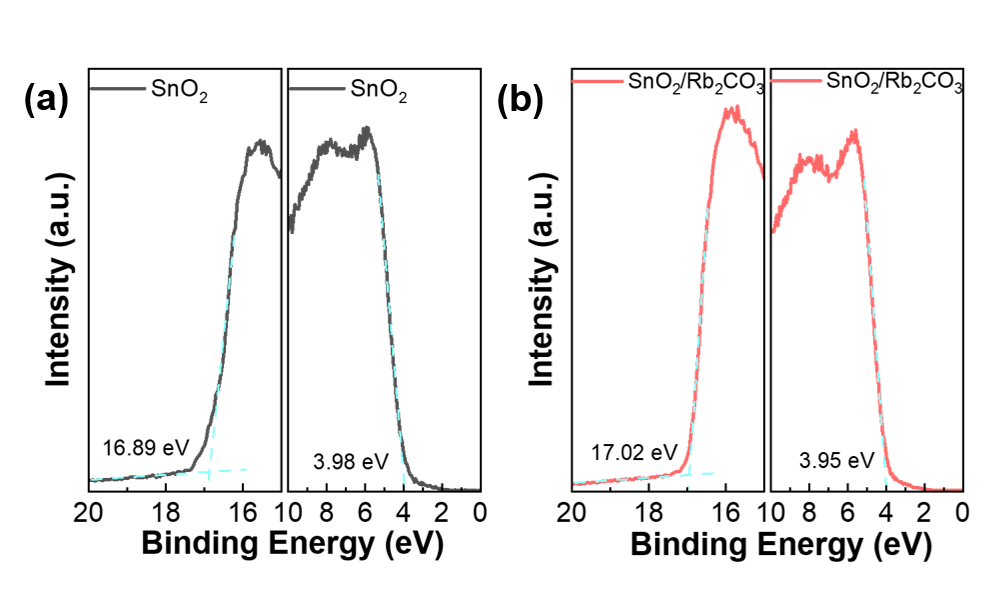


**Figure S20.** UPS spectra of (a) SnO_2_ and (b) SnO_2_/Rb_2_CO_3_ film. The dipole effect of Rb_2_CO_3_ led to a reduction in the work function (*W_F_*) of the SnO_2_ film from 4.33 eV to 4.20 eV, shifting the valence band maximum from –8.31 eV to –8.15 eV, and the conduction band minimum (CBM) from –4.49 eV to –4.35 eV.


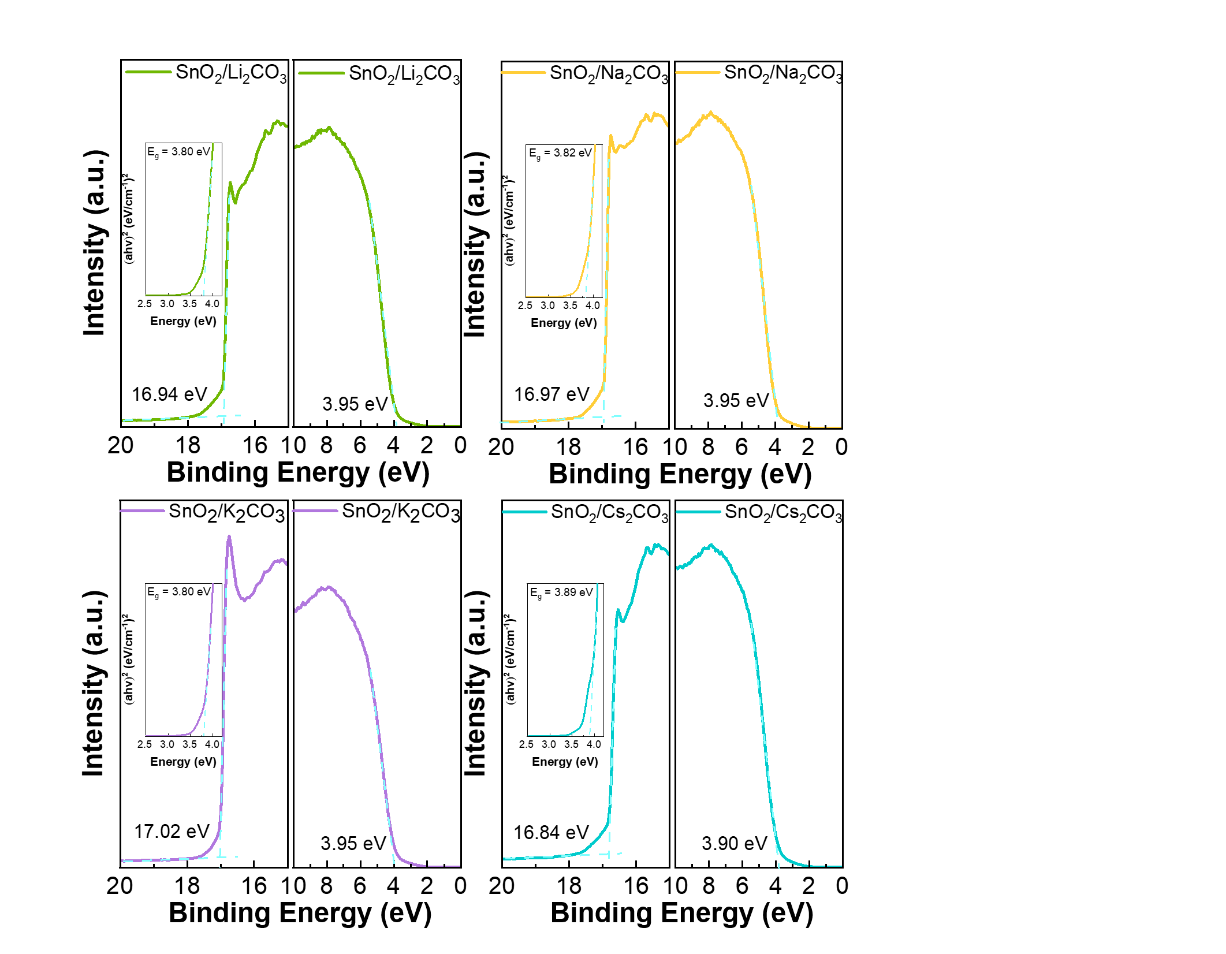


**Figure S21.** UPS spectra of SnO_2_ films with and without A_2_CO_3_ treatment, with insets showing the Tauc plot of the corresponding films. The *W_F_* values of the A_2_CO_3_-treated films decreased from 4.33 eV for control to 4.28 eV for Li_2_CO_3_-, 4.25 eV for Na_2_CO_3_-, 4.20 for K_2_CO_3_-, and 4.36 eV for Cs_2_CO_3_-treated SnO_2_ films.


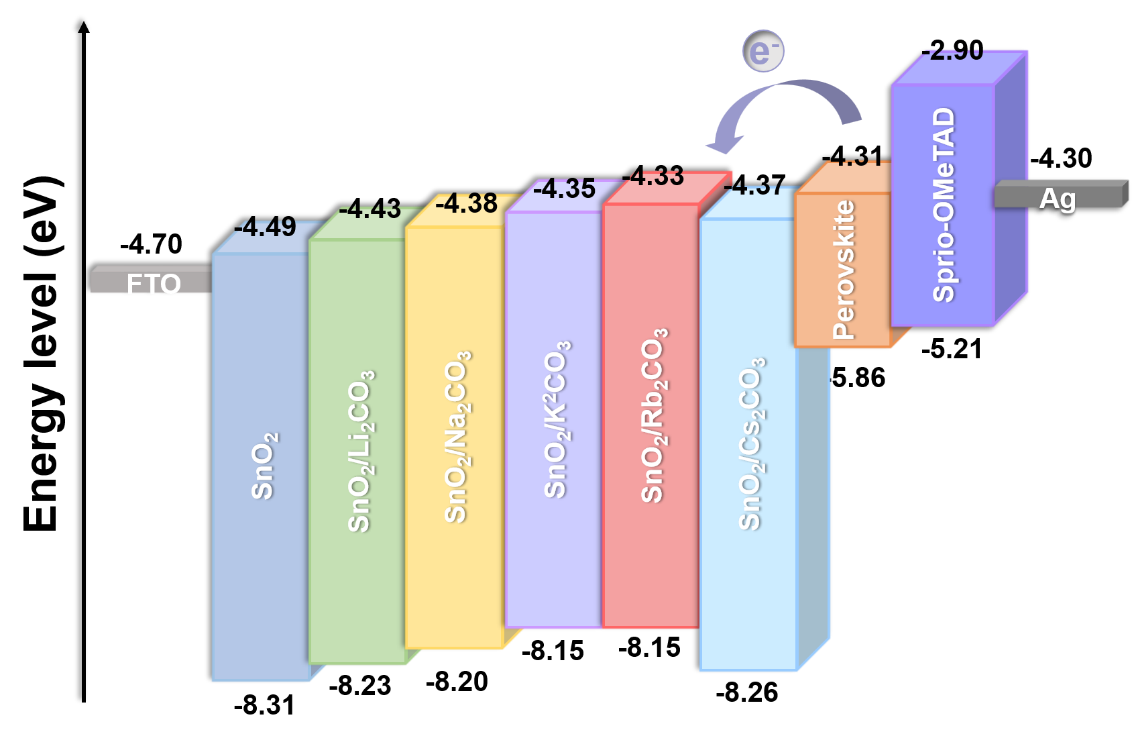


**Figure S22.** Energy level diagram of PSCs with and without A_2_CO_3_ treatment. The decreased *W_F_* resulting from the dipole moment of A_2_CO_3_ reduces the energy level barrier, facilitating electron extraction from the CBM of perovskite films, enhancing *V_OC_* and fill factor (*FF*).


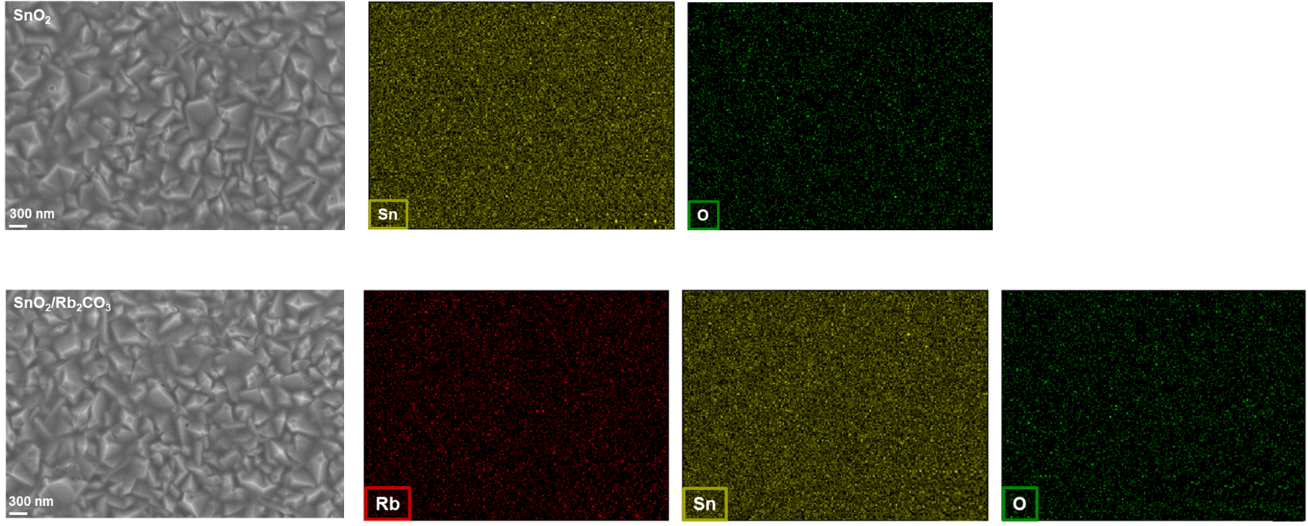


**Figure S23.** FE-SEM images and EDS mapping of SnO_2_ and SnO_2_/Rb_2_CO_3_ films on FTO substrate. The Rb, Sn, and O elements are uniformly distributed on SnO_2_ film without agglomeration.


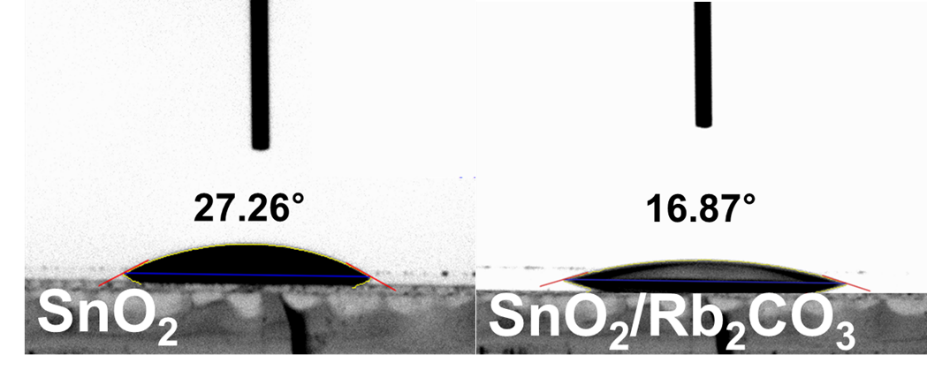


**Figure S24.** The water contact angle of SnO_2_ and SnO_2_/Rb_2_CO_3_ films.


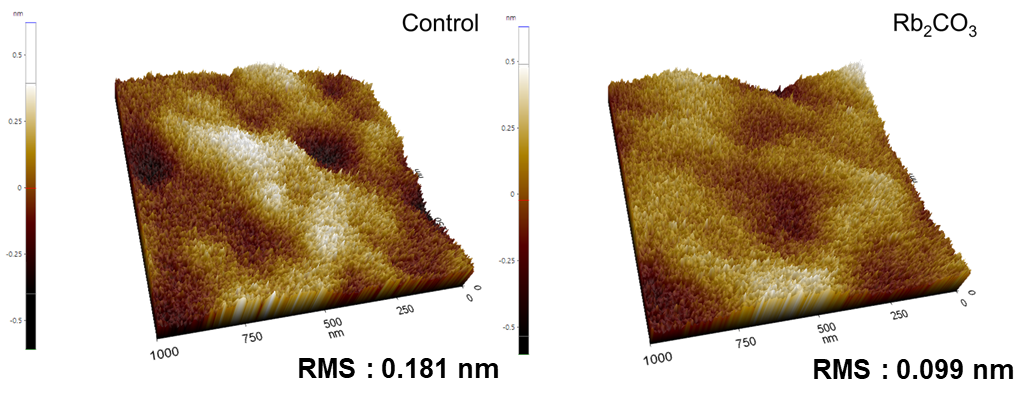


**Figure S25.** AFM images of SnO_2_ film with and without Rb_2_CO_3_ on Si wafer. The AFM images reveal that the surface root-mean-square (RMS) roughness of the SnO_2_ film decreased from 0.181 nm to 0.099 nm upon Rb_2_CO_3_ treatment. The smoother surface contributes to better interfacial contact with the upper perovskite layer, leading to improved crystallization and enhanced charge extraction at the interface, ultimately providing a higher-quality perovskite film.

**
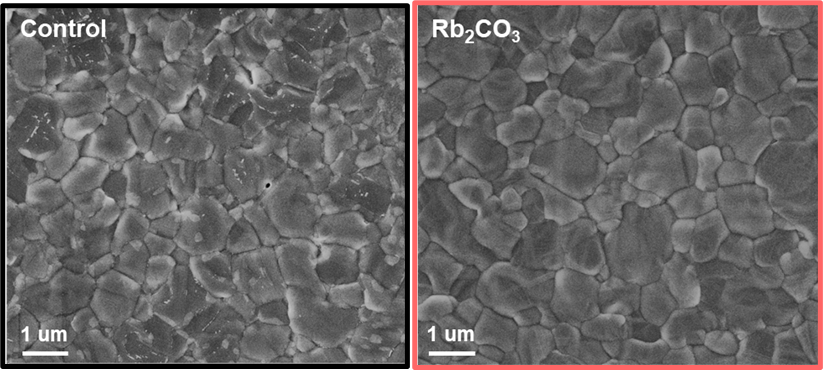
**

**Figure S26.** Top–view SEM images of top surface of control and Rb_2_CO_3_–treated perovskite films.


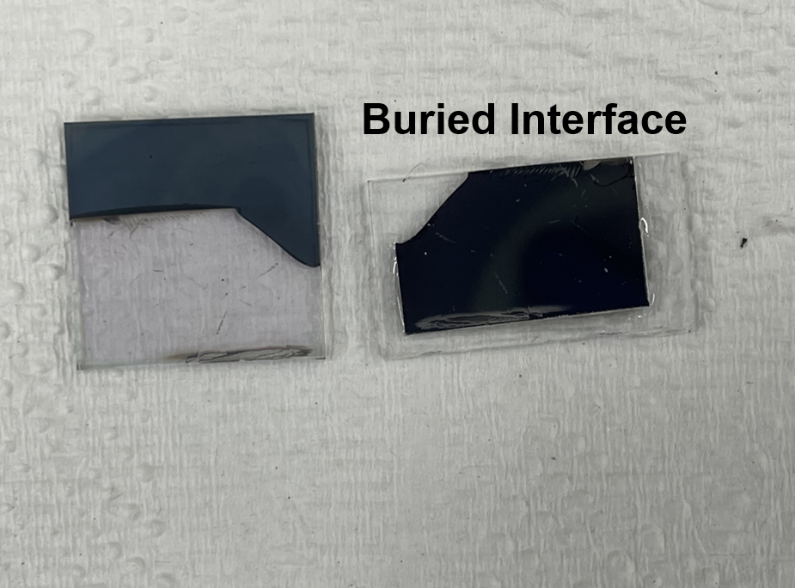


**Figure S27.** The photographs depict the bottom side of perovskite film. To facilitate the removal of the perovskite films from the FTO/SnO_2_/Rb_2_CO_3_ substrate, an UV-curable glue resin was applied onto the perovskite films and then covered with glass. Substrate and glass are detached to separate the perovskite films from the substrate, enabling the acquisition of the buried interface of perovskite.


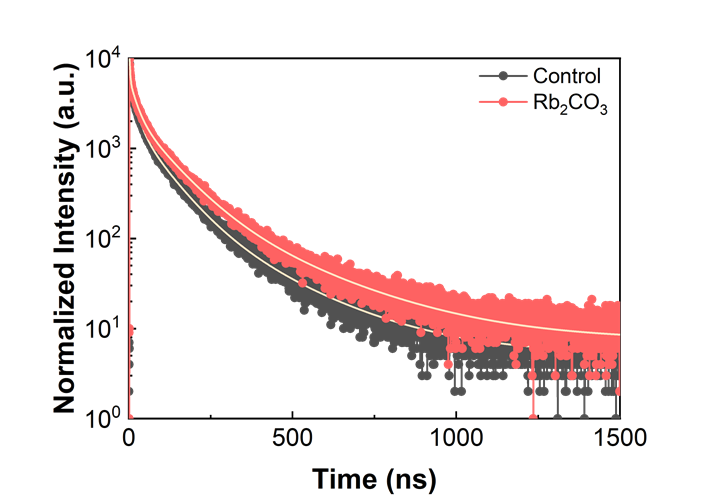


**Figure S28.** Normalized TRPL spectra of perovskite films with and without Rb_2_CO_3_ interlayer.


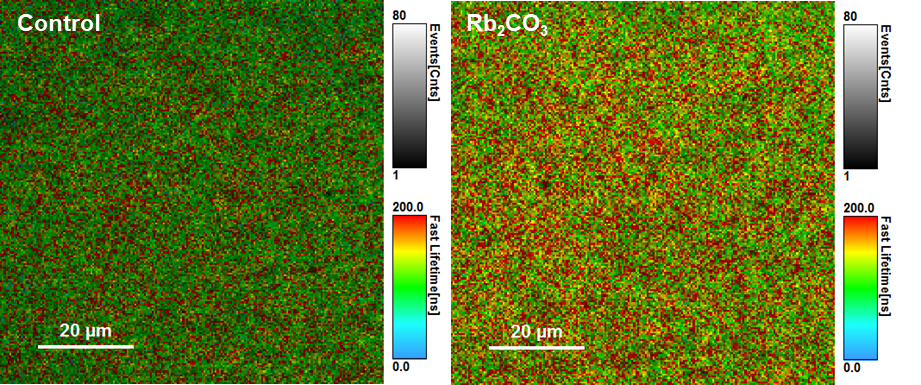


**Figure S29.** Fluorescence lifetime images of perovskite films with and without Rb_2_CO_3_ interlayer.


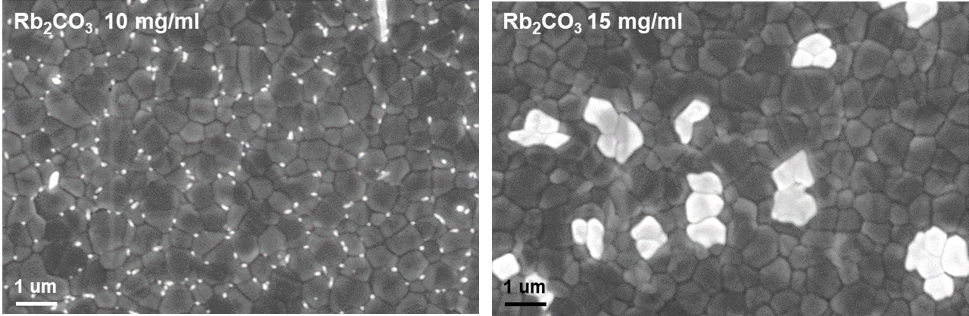


**Figure S30.** FE-SEM images of perovskite films with excess Rb_2_CO_3_ interlayer. The FE-SEM images show the presence of a bright impurity phase along grain boundaries, indicating a significant penetration of Rb^+^ into the perovskite film and the formation of a segregated Rb-rich phase, specifically photoinactive RbPbI_3._^[10]^


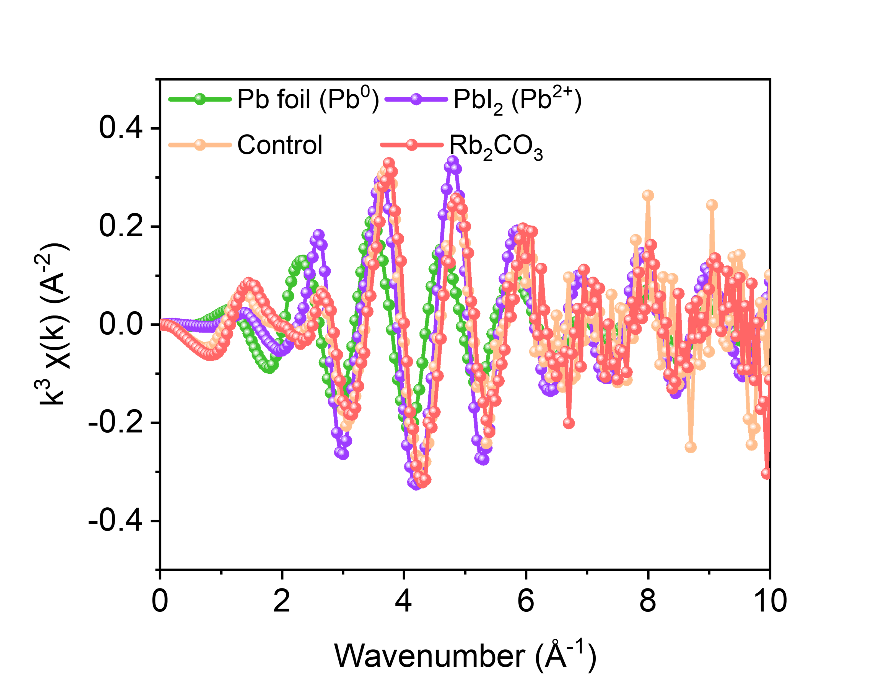


**Figure S31.** *k^3^*-weighted oscillation spectra in *k*-space for control and Rb_2_CO_3_-treated perovskite films.


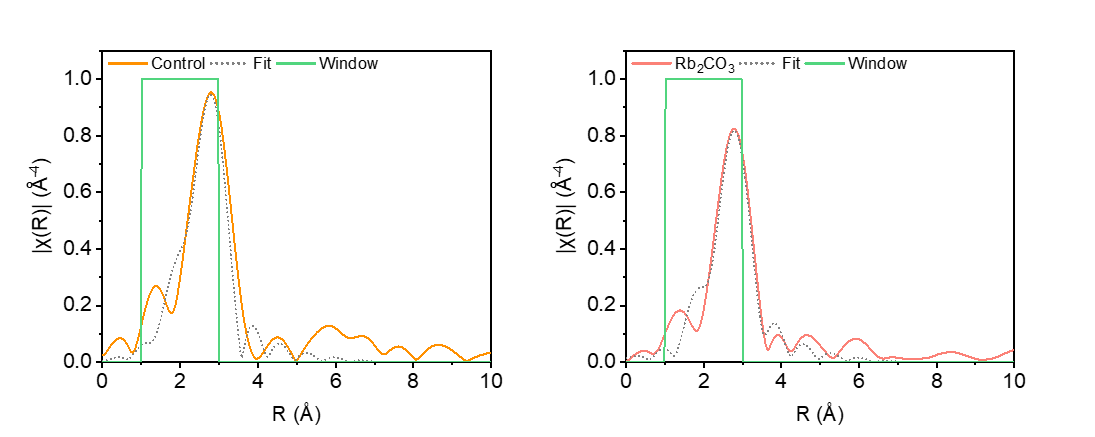


**Figure S32.** FT-EXAFS spectra in *R*-space, fitted using the ATHENA and ARTEMIS programs of the IFEFFIT package.


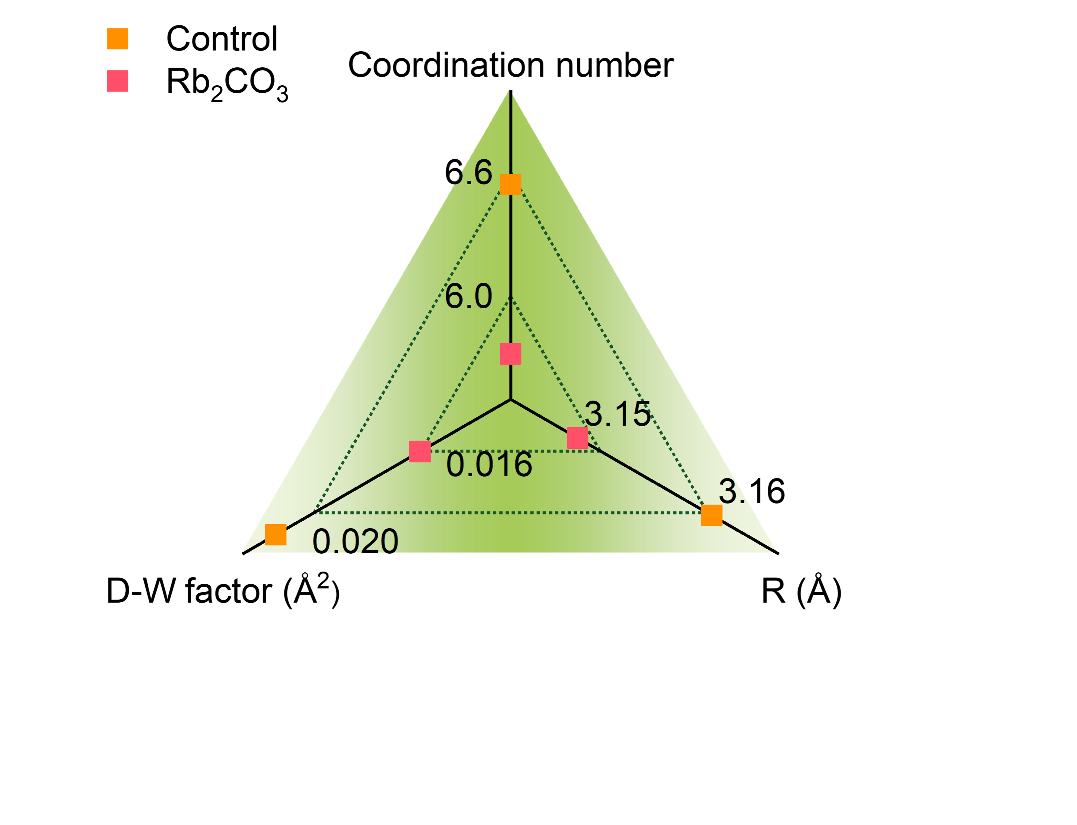


**Figure S33.** Factors from EXAFS for control and Rb_2_CO_3_-treated perovskite films


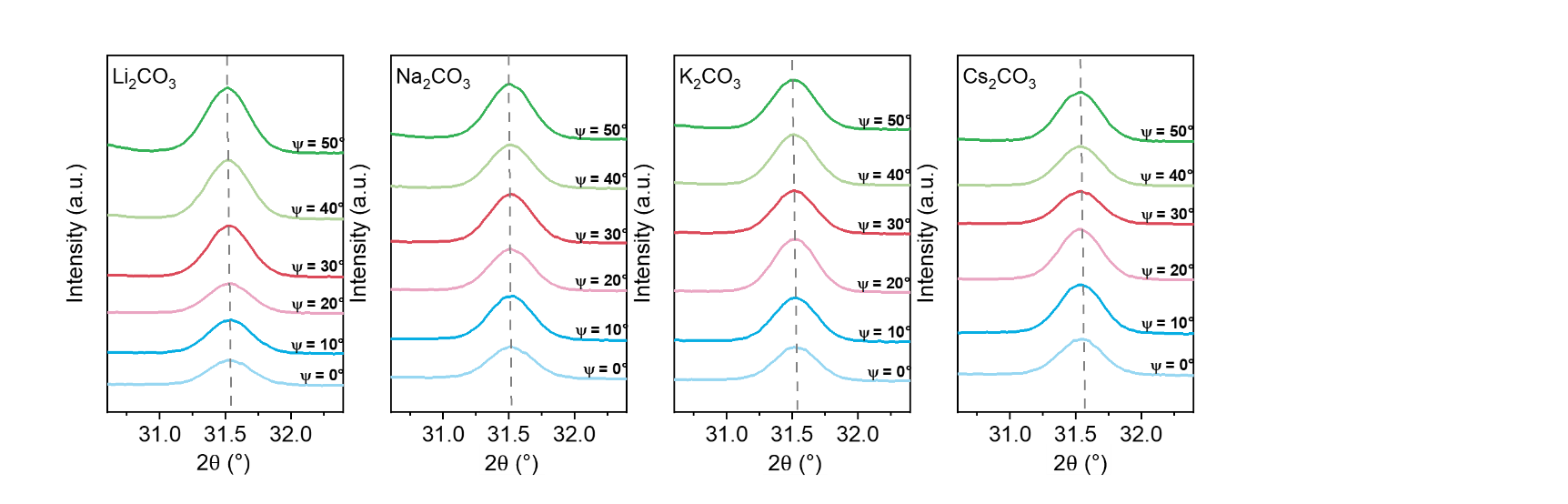


**Figure S34.** GIXRD spectra with different ψ values from 0° to 50° of Li_2_CO_3_-, Na_2_CO_3_-, K_2_CO_3_-, Cs_2_CO_3_-treated perovskite films.


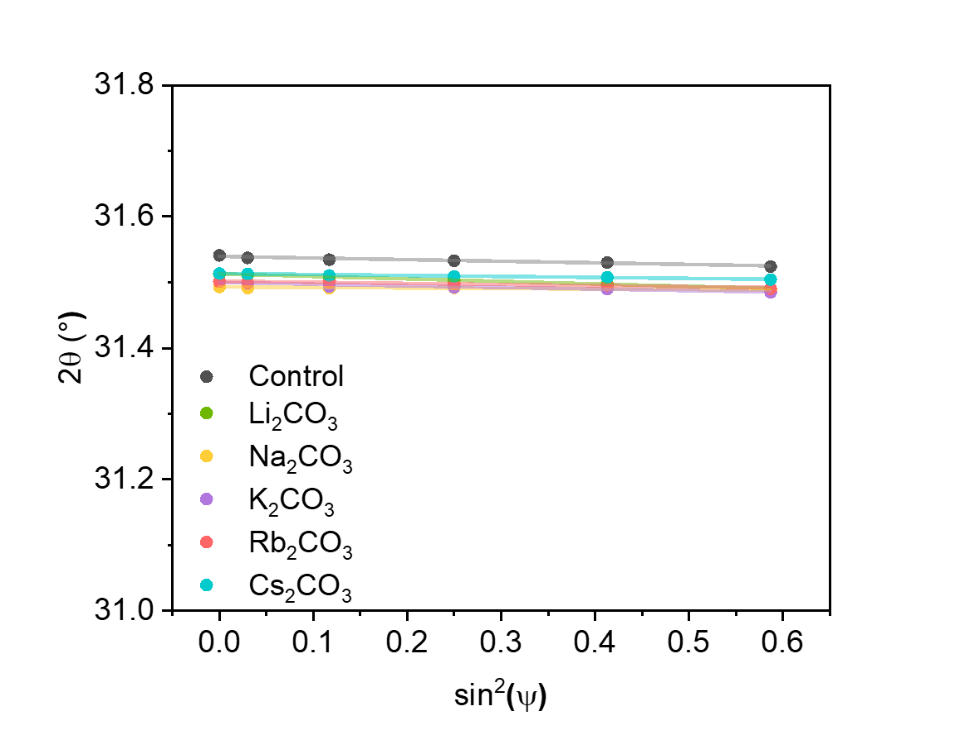


**Figure S35.** Linear fit of 2*θ*-sin^2^(ψ) of Li_2_CO_3_-, Na_2_CO_3_-, K_2_CO_3_-, Cs_2_CO_3_-treated perovskite films.

**Note S2:** Residual stress on the influence of A_2_CO_3_ treatment

To investigate the residual stress in perovskite films treated with A_2_CO_3_, additional GIXRD measurements were performed. As the tilt angle increased, it was observed that the peak of the (210) plane shifted leftward, indicating an increase in the lattice volume of the perovskite film according to Bragg's equation, signifying the presence of tensile stress. Furthermore, to quantify the residual stress, the 2*θ*–sin^2^(ψ) graph was fitted. According to Figure S35, the slopes for the control film, Li_2_CO_3_-, Na_2_CO_3_-, K_2_CO_3_-, and Cs_2_CO_3_-treated film were -0.2466, -0.03794, -0.00775, -0.2466, and -0.01476, respectively. Moreover, applying the formula for residual stress calculation yielded residual stress values of control film (6.5 ± 0.8 MPa), Li_2_CO_3_- (10.0 ± 0.4 MPa), Na_2_CO_3_- (2.0 ± 0.4 MPa), K_2_CO_3_- (6.5 ± 0.8 MPa), Rb_2_CO_3_- (4.4 ± 0.6 MPa), and Cs_2_CO_3_-tread film (3.9 ± 0.3 MPa).

Treating the interface with A_2_CO_3_, except for Li_2_CO_3_, leads to a reduction in residual stress of the perovskite films. This reduction in residual stress can be attributed to the relatively high thermal expansion coefficient of A_2_CO_3_, approximately 22.07 × 10^-5^ K^-1^, which helps mitigate the significant thermal expansion mismatch between SnO_2_ and the perovskite.^[11]^ Additionally, the presence of A_2_CO_3_ at the interface enables the formation of A-O-Sn bonds, which mitigates the formation of V_O_ defects, thereby alleviating interface stress and influencing the crystal growth of the perovskite film. A_2_CO_3_ at the interface can also bind with undercoordinated Pb, promoting the growth of the perovskite and enhancing its crystallinity.^[12]^ This alleviated residual stress plays a role in delaying the α to δ phase transition and improving the stability of the perovskite phase.^[13]^ Meanwhile, Li^+^ exhibits a faster migration ability within the perovskite films compared to other A-cations. This presence of Li^+^ at grain boundaries or interstitial sites promotes expansion of the perovskite lattice volume, resulting in slightly higher residual stress compared to the control film.^[14,15]^





**Figure S36.** Distribution of hysteresis index of PSCs with and without Rb_2_CO_3­_.





**Figure S37**. EQE spectra and integrated *J_SC_* of PSCs based on SnO_2_ and SnO_2_/Rb_2_CO_3_ films.


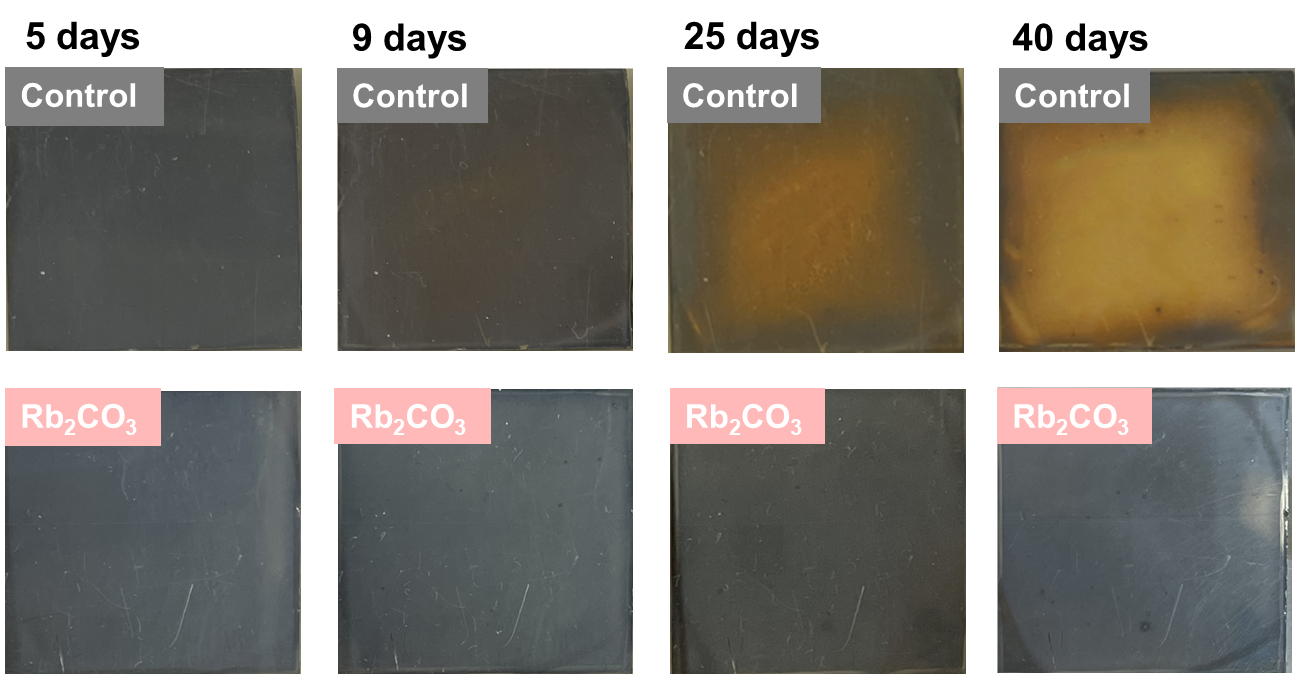


**Figure S38.** Photographs of control and Rb_2_CO_3_-treated perovskite films stored in ambient air (>50% RH) under illumination.

**Table S1.** Calculated surface energy of different termination in SnO_2_ (110) surface. For termination, a stoichiometric surface with a half terminal O was found to be energetically lower than non-stoichiometric O-terminated or Sn-terminated surfaces.

|  | Stoichiometric (110) | O-terminated (110) | Sn-terminated (110) |
| --- | --- | --- | --- |
| Surface energy (J/m^2^) | 1.018 | 2.417 | 2.692 |

**Table S2.** Binding energies from Sn 3d XPS spectrum of SnO_2_ films and SnO_2_/A_2_CO_3_ films.

|  | Sn 3d_5/2_ [eV] | Sn 3d_3/2_ [eV] |
| --- | --- | --- |
| Control | 487.1 | 495.5 |
| Li_2_CO_3_ | 486.6 | 495.0 |
| Na_2_CO_3_ | 486.5 | 495.0 |
| K_2_CO_3_ | 486.6 | 495.0 |
| Rb_2_CO_3_ | 486.2 | 494.6 |
| Cs_2_CO_3_ | 486.4 | 494.9 |

**Table S3.** Photovoltaic parameters of the champion PSCs with and without A_2_CO_3_ treatment at SnO_2_/perovskite interface.

| Sample | *J_SC_* [mA/cm^2^] | *V_OC_* [V] | *FF* [%] | *PCE* [%] |
| --- | --- | --- | --- | --- |
| Control | 23.22 | 1.093 | 79.27 | 20.11 |
| Li_2_CO_3_ | 22.61 | 1.119 | 80.43 | 20.36 |
| Na_2_CO_3_ | 23.33 | 1.125 | 78.82 | 20.69 |
| K_2_CO_3_ | 23.14 | 1.138 | 81.67 | 21.50 |
| Rb_2_CO_3_ | 23.21 | 1.149 | 82.84 | 22.10 |
| Cs_2_CO_3_ | 23.18 | 1.136 | 79.84 | 21.01 |

**Table S4.** Structural parameters of Pb–I bond from fitting to Fourier transformed EXAFS data.^a)^

| Sample | Coordination number (CN) | *D–W* factor [σ^2^] | *R* [Å] | *R* factor |
| --- | --- | --- | --- | --- |
| Control | 6.54±0.327 | 0.0213±0.001 | 3.16±0.015 | 0.003 |
| Rb_2_CO_3_ | 5.72±0.523 | 0.0167±0.001 | 3.15±0.015 | 0.003 |

^a)^ The CN of Pb atoms was determined using the amplitude reduction factor (S_0_^2^). Accordingly, the CN of the Pb atoms in the perovskite film was subsequently fitted by referring to the S_0_^2^ obtained from pure PbI_2_ powder.^[16]^ The degree of certainty of fit was evaluated using the *R*-factor.

**Table S5.** Photovoltaic parameters of perovskite solar cells under different scan directions.

| Sample | Scan direction | *J_SC_* [mA/cm^2^] | *V_OC_* [V] | *FF* [%] | *PCE* [%] | Hysteresis Index |
| --- | --- | --- | --- | --- | --- | --- |
| Control | Forward | 23.22 | 1.093 | 79.27 | 20.11 | 0.021 |
|  | Reverse | 23.17 | 1.086 | 78.31 | 19.70 |  |
| Rb_2_CO_3_ | Forward | 23.21 | 1.149 | 82.84 | 22.10 | 0.013 |
|  | Reverse | 23.04 | 1.145 | 82.69 | 21.81 |  |

**Table S6.** Fitted parameters for electrical impedance spectroscopy data of control and Rb_2_CO_3_-treated PSCs obtained under dark conditions.

| Sample | *R_s_* (Ω) | *R_tr_* (Ω) | *R_rec_* (Ω) |
| --- | --- | --- | --- |
| Control | 18.03 | 8495 | 36280 |
| Rb_2_CO_3_ | 10.63 | 4373 | 57040 |

**References**

[1] S. Sun, F. H. Isikgor, Z. Deng, F. Wei, G. Kieslich, P. D. Bristowe, J. Ouyang, A. K. Cheetham, *ChemSusChem* **2017**, *10*, 3740.

[2] Y. Chen, Y. Lei, Y. Li, Y. Yu, J. Cai, M.-H. Chiu, R. Rao, Y. Gu, C. Wang, W. Choi, *Nature* **2020**, *577*, 209.

[3] G. Kresse, J. Furthmüller, *Phys. Rev. B* **1996**, *54*, 11169.

[4] P. E. Blöchl, *Phys. Rev. B* **1994**, *50*, 17953.

[5] J. P. Perdew, K. Burke, M. Ernzerhof, *Phys. Rev. Lett.* **1996**, *77*, 3865.

[6] S. Grimme, *J. Comput. Chem.* **2006**, *27*, 1787.

[7] M. Batzill, U. Diebold, *Prog. Surf. Sci.* **2005**, *79*, 47.

[8] F. Urbach, *Phys. Rev.* **1953**, *92*, 1324.

[9] B. Subedi, C. Li, C. Chen, D. Liu, M. M. Junda, Z. Song, Y. Yan, N. J. Podraza, *ACS Appl. Mater. Interface.* **2022**, *14*, 7796.

[10] M. Zhang, J. S. Yun, Q. Ma, J. Zheng, C. F. J. Lau, X. Deng, J. Kim, D. Kim, J. Seidel, M. A. Green, *ACS Energy Lett.* **2017**, *2*, 438.

[11] S. M. Hurt, R. A. Lange, *Geoch. Cosm. Act.* **2019**, *248*, 123.

[12] R. Xu, F. Pan, J. Chen, J. Li, Y. Yang, Y. Sun, X. Zhu, P. Li, X. Cao, J. Xi, *Adv. Mater.* **2024**, *36*, 2308039.

[13] C. Zhu, X. Niu, Y. Fu, N. Li, C. Hu, Y. Chen, X. He, G. Na, P. Liu, H. Zai, Y. Ge, Y. Lu, X. Ke, Y. Bai, S. Yang, P. Chen, Y. Li, M. Sui, L. Zhang, H. Zhou, Q. Chen, *Nat. Commun.* **2019**, *10*, 815.

[14] K. Wang, W. S. Subhani, Y. Wang, X. Zuo, H. Wang, L. Duan, S. Liu, *Adv. Mater.* **2019**, *31*, 1902037.

[15] J. Zhang, R. Chen, Y. Wu, M. Shang, Z. Zeng, Y. Zhang, Y. Zhu, L. Han, *Adv. Energy Mater.* **2018**, *8*, 1701981.

[16] D.–H. Kang, Y.–J. Park, Y.–S. Jeon, N.–G. Park, *J. Energy Chem.* **2022**, *67*, 549.
